# Antibiotic-induced gut microbiota dysbiosis and bile acid metabolism: implications for intestinal health, immune regulation, and disease susceptibility

**DOI:** 10.3389/fmicb.2026.1918510

**Published:** 2026-08-25

**Authors:** Tareq Nayef AlRamadneh, Jasur Rizaev, Malathi Hanumanthayya, Maryam Abdul Al-Hussein, Ahmed Faisal Mutee, Mukhammadali Buriev, Nasiba Jumaniyazova, Shefali

**Affiliations:** 1 Faculty of Allied Medical Sciences, Hourani Center for Applied Scientific Research, Al-Ahliyya Amman University, Amman, Jordan; 2 Department of Public Health and Healthcare Management, Samarkand State Medical University, Samarkand, Uzbekistan; 3 Department of Biotechnology and Genetics, School of Sciences, JAIN (Deemed to Be University), Bengaluru, India; 4 Department of Medical Laboratory Technologies, College of Medical Technologies, The Islamic University, Najaf, Iraq; 5 College of Pharmacy, Alnoor University, Mosul, Iraq; 6 Department of Medicine, Termez University of Economics and Service, Termez, Uzbekistan; 7 Department of Psychology, Mamun University, Khiva, Uzbekistan; 8 University Institute of Pharma Sciences, Chandigarh University, Mohali, India

**Keywords:** antibiotic, bile acids, *C. difficile*, dysbiosis, FXR, gut microbiota, gut–brain axis, immune regulation

## Abstract

Intestinal microorganisms regulate bile acid (BA) homeostasis through bile salt hydrolase (BSH)-mediated deconjugation and a series of biotransformation reactions that generate secondary BAs with potent signaling functions. Antibiotic exposure profoundly disrupts these microbial processes, leading to alterations in BA composition, diversity, and enterohepatic circulation. Recent multi-omics studies have demonstrated that antibiotics reduce the abundance of key BA-transforming bacteria, including members of the Lachnospiraceae, Ruminococcaceae, Lactobacillaceae, Clostridiaceae, and Bacteroidaceae families, resulting in decreased BSH activity, depletion of secondary BAs, accumulation of conjugated primary BAs, and impaired BA signaling. Mechanistically, antibiotic-induced BA dysregulation affects several host regulatory pathways, including the farnesoid X receptor (FXR), Takeda G protein-coupled receptor 5 (TGR5), nucleotide-binding domain, leucine-rich–containing family, pyrin domain–containing-3 (NLRP3) inflammasome signaling, glucagon-like peptide-1 (GLP-1) secretion, and mammalian target of rapamycin (mTOR) signaling. Emerging evidence further suggests that BA metabolites serve as critical mediators linking antibiotic-induced dysbiosis with host hemostasis remodeling, T helper (Th)17-cell differentiation, and astrocyte activation, among others. These alterations contribute to diverse pathological outcomes, including loss of colonization resistance against *Clostridium difficile*, cholestatic liver injury, metabolic dysfunction, immune dysregulation, autoimmune inflammation, and gut–brain axis dysfunction. Conversely, selective manipulation of microbial BA metabolism may provide therapeutic benefits in specific disease contexts. Inhibition of microbial BSH activity by gentamicin alleviates experimental cholestasis, while microbiota-preserving narrow-spectrum antibiotics maintain secondary BA production and improve resistance to recurrent *C. difficile* infection. This review summarizes current evidence regarding the effects of antibiotics on microbial and molecular mechanisms driving BA imbalance, and discusses the implications of altered BA signaling for intestinal, hepatic, metabolic, immune, and neurological health.

## 1 Introduction

Ever since their invention at the turn of the twentieth century, antibiotics have been considered efficient agents for fighting against infectious diseases, providing numerous clinical advantages and temporary protection against a number of infections (Khan et al., 2019). However, their misuse, overuse, and prolonged usage can lead to serious problems in the management of chronic diseases. Among other issues, their usage can provoke dysbiosis, resulting in various disease states in the host body (Kuribayashi et al., 2012). In particular, antibiotics can impair the gut microbiota by influencing the ratio between the dominant phyla of bacteria (Firmicutes, Bacteroidetes, and Proteobacteria) (Antonopoulos et al., 2009). As a result, the imbalance of these bacterial populations can be seen as a decrease in the ratio between Firmicutes and Bacteroidetes; this ratio decreases due to the fact that Bacteroidetes grow faster than Firmicutes under antibiotic selective pressure (Panda et al., 2014).

The gut flora, along with the metabolites that they secrete, particularly bile acids (BAs), play an important role in many physiologic processes like nutrient absorption, metabolic modulation, maintaining homeostasis, and immunoprotection (Zhao et al., 2025). By acting on certain receptors in the liver and intestine, the BAs impact different metabolic processes in the body, which include glucose and lipid metabolism, energy metabolism, and inflammatory signaling (Zhao et al., 2025).

The utilization of animal models has allowed us to gain much understanding of how the gut microbiome contributes to the generation of various metabolites (Khan et al., 2019). The comparisons made between conventionally housed animals exposed to microbes, germ-free (GF) animals, and animals that underwent antimicrobial therapy have been extensively performed in order to shed some light on how the gut microbiota influences metabolite synthesis and regulation (Sun et al., 2013; Li Y. et al., 2017). For instance, it was found that GF mice possessed higher concentrations of conjugated BAs such as taurocholic acid and tauro-β-muricholic acid along with lower concentrations of secondary BAs in the lumen compared to conventionally housed animals, pointing out the pivotal role of the gut microbiota in BA metabolism (Sayin et al., 2013). In addition, studies performed in the context of humans and animal models showed that antibiotic treatment is highly correlated with impaired BA metabolism (Sun et al., 2013; Schulfer et al., 2018). For example, experiments performed in mice have highlighted the role of intestinal microflora in regulating the FXR–FGF signaling pathway (Young and Schmidt, 2004; Schulfer et al., 2018). The administration of ampicillin to mice was followed by enhanced concentrations of conjugated BAs (taurocholic acid and tauro-β-muricholic acid) and lower concentrations of unconjugated BAs (CA, β-muricholic acid) (Hopkins and Macfarlane, 2002; Chang et al., 2008).

Consistent results have been obtained for azithromycin, clarithromycin, erythromycin, telithromycin, amoxicillin, flucloxacillin, and florfenicol administration in rats, leading to an elevation in plasma concentrations of taurocholic acid and simultaneous lowering of plasma concentrations of unconjugated primary and secondary BAs, as well as taurine-conjugated and unconjugated BA species (Sun et al., 2013; Hamoud et al., 2018). On the other hand, a study on humans has indicated a reduction in fecal secondary BA concentration accompanied by an increase in primary BA after vancomycin administration (Vrieze et al., 2014). Ten-day cefoperazone treatment also affected the composition of BAs via lowering the secondary-to-primary BA ratio and increasing conjugated BA concentrations (Theriot et al., 2014). Many of the recent reviews have extensively elaborated on the interplay between the gut microbiota and BA metabolism; most of them have emphasized the physiological regulation of BA homeostasis and/or its role in certain diseases. Of note, the present review highlights the impact of antibiotics on the disturbance of the microbiome-BA metabolism as the key mechanism underlying host dysfunction due to dysbiosis. The review covers recent developments that explain how antibiotics lead to changes in the population of BA-transforming bacteria and their metabolic activities, thereby affecting BA profile, signaling through the farnesoid X receptor (FXR), Takeda G protein-coupled receptor 5 (TGR5), NOD-, LRR- and pyrin domain-containing protein 3 (NLRP3), glucagon-like peptide-1 (GLP-1), and mammalian target of rapamycin (mTOR) receptors, and various intestinal, hepatic, metabolic, immunological, neurological, and cancer-related disorders. In addition, therapeutic interventions targeting the restoration of BA homeostasis following antibiotic treatment by means of preserving the microbiota, fecal microbiota transplantation, probiotics, and manipulating bacterial BA metabolism are also covered.

## 2 Bile acids: from hepatic synthesis to enterohepatic circulation

**FIGURE 1:**
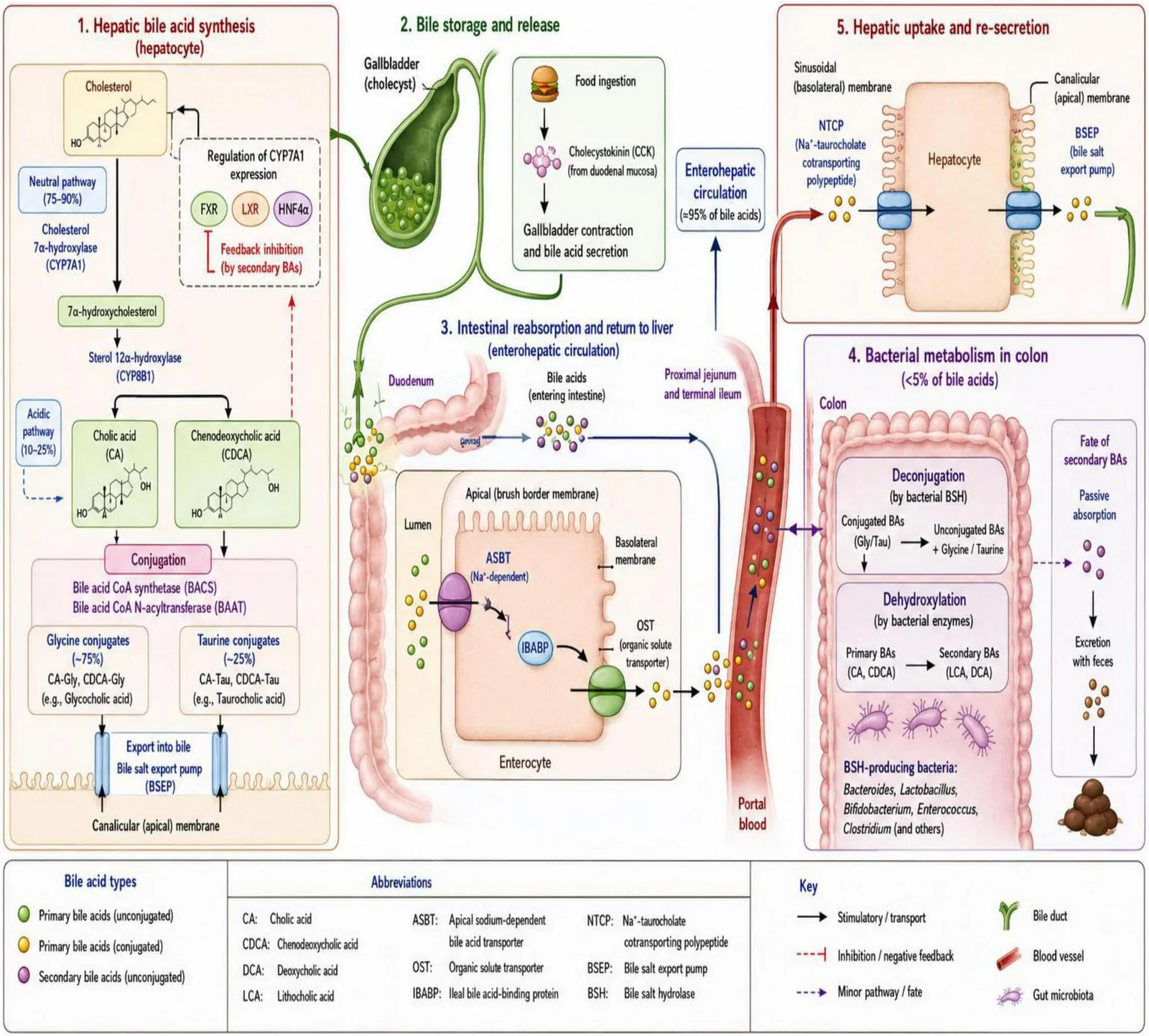
Overview of bile acid synthesis, microbial transformation, and enterohepatic circulation. Primary bile acids (BAs) are synthesized from cholesterol in hepatocytes through the neutral pathway (predominantly mediated by CYP7A1) and the acidic pathway. The activity of CYP8B1 determines the ratio of cholic acid (CA) to chenodeoxycholic acid (CDCA). Newly synthesized BAs are conjugated with glycine or taurine by BA CoA synthetase (BACS) and BA CoA (BAAT), producing more water-soluble conjugated BAs. Conjugated BAs are secreted into bile through the bile salt export pump (BSEP), stored in the gallbladder, and released into the small intestine following food intake and cholecystokinin (CCK)-induced gallbladder contraction. Approximately 95% of BAs are reabsorbed in the proximal jejunum and terminal ileum via the apical sodium-dependent BA transporter (ASBT). Within enterocytes, BAs bind to ileal BA-binding protein (IBABP) and are exported across the basolateral membrane by the organic solute transporter (OST). Reabsorbed BAs return to the liver through the portal circulation and are taken up by hepatocytes via the Na+-taurocholate co-transporting polypeptide (NTCP), completing the enterohepatic circulation. Less than 5% of BAs escape ileal reabsorption and enter the colon, where gut microbial enzymes, particularly bile salt hydrolases (BSHs), catalyze deconjugation reactions. Subsequent bacterial transformations, including 7α-dehydroxylation, convert primary BAs into secondary BAs such as deoxycholic acid (DCA) and lithocholic acid (LCA). Secondary BAs may be passively absorbed and returned to the liver or excreted in feces. Secondary BAs also regulate hepatic BA synthesis through feedback activation of the farnesoid X receptor (FXR), thereby contributing to the maintenance of BA homeostasis and systemic lipid metabolism.

The BAs represent steroid-based compounds that are generally classified into two types depending upon their location of synthesis (Wang et al., 2025). The primary BA synthesis occurs in the liver from the conversion of cholesterol by enzymatic action, and is primarily composed of cholic acid (CA), chenodeoxycholic acid (CDCA), and glycine/taurine conjugates of these BAs (Figure 1; Wang et al., 2025). After their secretion into the intestine, they undergo metabolism due to the action of microorganisms present in the gut to form secondary BAs like deoxycholic acid (DCA) and lithocholic acid (LCA; Rimal et al., 2024). Hepatic BA synthesis constitutes the primary mechanism responsible for cholesterol catabolism and plays an important role in regulating the composition of BAs in circulation. Through its impact on BA availability and composition, this process influences gut microbiota and modulates BA-dependent host metabolic signaling (Di Ciaula et al., 2018). BA biosynthesis proceeds via two pathways, namely, the neutral and acidic ones. Both these routes are regulated according to the current metabolic state and inflammatory conditions (Di Ciaula et al., 2018).

The neutral pathway is responsible for BA biosynthesis at 75%–90%, and its starting step involves the hydroxylation of cholesterol by the enzyme cholesterol 7α-hydroxylase coded by CYP7A1. CYP7A1 is considered the rate-limiting enzyme in BA synthesis, and, accordingly, its expression is controlled by different transcription factors like FXR, liver X receptor (LXR), and hepatocyte nuclear factor 4α (HNF4α; Ramírez-Pérez et al., 2018). Metabolically produced secondary BAs act as feedback regulators of this process, as they exhibit a higher affinity to FXR than primary BAs. One important branch point in BA synthesis is the action of the enzyme sterol 12α-hydroxylase (CYP8B1), which regulates the ratio between the production of CA and CDCA. Primary BAs produced by the hepatocytes are subsequently conjugated mainly with glycine (approximately 75%) and sometimes taurine (25%). This reaction is catalyzed by the enzymes BA CoA synthetase (BACS) and BA CoA acid N-acyltransferase (BAAT; Kang et al., 2025). Bacterial transformation of conjugated BAs can only proceed after their deconjugation. Conjugation serves as a mechanism to reduce toxicity, increase aqueous solubility of BAs, and inhibit passive BA absorption prior to reaching the gut; at the same time, conjugation provides the substrate necessary for microbial bile salt hydrolase (BSH) action. Depending on the individual, the proportion of glycine- vs. taurine-conjugated BAs varies greatly, which in turn determines the availability of substrate for their further deconjugation and metabolism by bacteria (Takada and Himmerich, 2021; Harnisch et al., 2023).

Deconjugation of BAs within the gut is accomplished by a variety of microbial enzymes, among which BSHs make up the largest fraction of enzymatically active proteins. In particular, BSHs hydrolyze the amide bond between primary BAs and glycine or taurine, yielding unconjugated BAs along with free amino acids (Mohanty et al., 2024). Being the mandatory prerequisite for almost any other metabolic modification of BAs performed by bacteria, BSH activity plays the pivotal role in the BA metabolism potential of the gut microbiome (Wise and Cummings, 2023). Bacterial genes coding BSH are widespread within various bacteria of *Bacteroides*, *Lactobacillus*, *Bifidobacterium*, *Enterococcus*, and *Clostridium* taxa. In addition, many species possess several paralogs of BSH with specific substrate preferences (Núñez-Sánchez et al., 2022). The metabolic robustness provided by such enzyme diversity allows efficient bacterial BA conversion to take place regardless of changes in bacterial composition. The most common primary BAs are conjugated with taurine or glycine, which enhances their water-solubility while at the same time decreasing their cytotoxic effects (Chiang and Ferrell, 2019). Once the conjugation step is completed, CA and CDCA are exported into bile through the activity of the bile salt export pump (BSEP) localized in the canalicular (apical) membrane of hepatocytes, after which these molecules are temporarily stored in the gallbladder (cholecyst) (Wahlström et al., 2016). In response to food ingestion, cholecystokinin produced by the duodenal mucosa induces gallbladder contraction leading to BA secretion (Li and Chiang, 2014).

In the proximal jejunum and terminal ileum, the vast majority of BAs, accounting for up to 95%, are taken back up by enterocytes through ASBT localized within the brush border membrane (Wahlström et al., 2016). In the cytoplasm of enterocytes, the intracellular transportation toward the basolateral membrane of BAs occurs due to their binding with ileal BA-binding protein (IBABP). Subsequently, BAs are exported into the bloodstream by organic solute transporters (OST) localized within the basolateral membrane of enterocytes (Jia et al., 2018). The recirculated BAs are carried into the liver with the portal blood flow and enter the hepatocyte cytoplasm via sodium taurocholate co-transporting polypeptide (NTCP), which is essential for the transport of conjugated BAs (Jia et al., 2018). Such a constant cycle of reabsorption of the recirculating BAs in the intestine and their release in the liver is called enterohepatic circulation and is responsible for the regulation of feedback inhibition of BA synthesis as well as systemic lipid homeostasis (Chiang, 2009; Marra and Svegliati-Baroni, 2018). The remaining less than 5% of BAs cannot be taken up by enterocytes and travel further down to the colon (Poland and Flynn, 2021). There, a portion of the BA pool is metabolized in the gut by deconjugation and dehydroxylation by bacterial enzymes to secondary BAs such as LCA and DCA. Secondary BAs may either be passively absorbed or excreted with feces (Kwong et al., 2015; Arab et al., 2017).

## 3 Antibiotics and microbiota structure and function

In recent years, more attention has been paid to the influence of antibiotics on taxonomical structure of the fecal microbiota, as well as their effects on the diversity and abundance of antibiotic resistance genes (Modi et al., 2014). At the same time, fewer studies addressed the impact of antibiotics on clinically relevant functions of the gut microbiome, including the consequences of the related changes for the host. One of the first reported effects of antibiotic administration was the disturbance of colonization resistance, which was called competitive exclusion at that time (Miller et al., 1956; Bohnhoff and Miller, 1962). Disruption of this defense mechanism results in higher sensitivity of the gut microbiome to colonization by pathogens, including *Salmonella*. Resistance to pathogenic colonization depends on both resource-based competition and direct microbial antagonism. It is worth adding that immune stimulation, as well as antimicrobial effector molecule production, plays its role too (Buffie and Pamer, 2013). The change of gut microbial composition induced by antibiotics can strongly affect the balance of nutrients and species interactions within the community. Recently, increased host-derived free sialic acid production during treatment with antibiotics was linked to increased colonization by opportunistic pathogens, such as *Salmonella Typhimurium* and *Clostridium difficile* (Ng et al., 2013).

Antibiotics, which demonstrate high efficacy against anaerobes, such as clindamycin, usually result in longer disturbances of gut microbial composition (Jernberg et al., 2005, 2007). Jakobsson et al. (2010) used a combination of clarithromycin, metronidazole, and omeprazole for a 7-day therapy; they reported profound changes in both pharyngeal and fecal microbiota composition, partial recovery of pharyngeal flora, and persisting microbiota disturbances up to four years after exposure. Even in the case of the antibiotic with weak activity toward anaerobes, such as ciprofloxacin, some effect on the gut microbiome composition could be expected. Dethlefsen et al. (2008) showed that 5-day treatment with ciprofloxacin changed the abundance of about one-third of gut bacterial taxa and significantly decreased the richness of the latter group within several days after treatment start. At the same time, although the reduction in diversity was similar for all participants, the duration and nature of microbiota recovery varied between individuals. Antibiotics can also induce damage in the form of structural and physiological disturbances in bacterial cells, thereby resulting in low enzymatic activity. This includes membrane disruption, disruption of membrane polarity, and reduced nucleic acid content, among others, in bacteria (Ferrer et al., 2017). In the process of treatment, susceptible bacteria tend to be displaced by resistant ones, which can survive and assist in sustaining some metabolic activities of the gut microbiome. This is usually referred to as functional redundancy and is characterized by the distribution of metabolic roles among different bacterial populations in order to maintain function in the presence of community changes (Ferrer et al., 2017). More specifically, antibiotic cocktails including cefazolin, ampicillin, and sulbactam may cause disturbances in the activity of glycoside hydrolases. The result of this activity is increased metabolism and absorption of carbohydrates in a manner that may lead to metabolic syndromes like obesity and type 2 diabetes (Ferrer et al., 2017). After antibiotics disrupt the gut microbiome, the bacteria may reorganize themselves into a different community that maintains the same functional ability as the initial one. Even in cases where resistant bacteria displace other types, there may not necessarily be equality in terms of efficiency because enzyme activities can still differ among functionally redundant classes.

Alterations in normal microbial colonization have always been shown to affect the normal development of the immune system (Patangia et al., 2022). This is due to the close association that exists between the two during their development. There has been evidence showing that the lack of gut microorganisms through the use of germ-free models results in changes in the physiological and immunological environment of the intestines. Some of these changes involve changes in the thickness and chemical composition of the mucus, decreased gastric motility, abnormal development and functionality of both intestinal epithelial and immune cells, as well as poor development of the immune system (Patangia et al., 2022). Antibiotic-induced alteration of gut microbes has also been shown to lead to the development of a TH1/TH2 ratio imbalance in favor of TH2, and this results in the development of atopy in addition to reducing the number of lymphocytes (Oyama et al., 2001). Cloxacillin-exposed neonatal rats were shown to experience alterations in microbial community structures in addition to changes in molecular profiles like decreased levels of major histocompatibility complex (MHC) class I and class II molecule expression genes, and defensin expression in Paneth cells. Molecular changes resulting from antibiotic treatment might affect the proper development of the mucosal barrier (Schumann et al., 2005). Mice exposed to antibiotics during prenatal development have been shown to experience an alteration in microbial communities’ colonization in addition to weakened CD8^+^ T cell responses toward viral infection, leading to poor immunity. Predisposition to infections in mice was also higher in the case of hygienic housing conditions (Gonzalez-Perez et al., 2016).

## 4 Some aspects of antibiotic effects on microbiota-derived bile acids

Antibiotics not only perturb the bacterial composition in the gut microbiota but, more importantly, interfere with its function as well, especially its role in regulating BA metabolism, which is one of the critical functions of the intestinal microbiota (Table 1). In particular, antibiotics decrease the population of bacteria that produce BSH and 7α-dehydroxylation enzymes and result in a reduced capacity for conversion of primary to secondary BAs, thereby causing increased levels of conjugated primary BAs along with reduced levels of secondary BAs. Such modifications in the BA pool lead to alterations in enterohepatic circulation and signaling via FXR, TGR5, GLP-1, NLRP3, and mTOR. Therefore, antibiotic-induced disruption in BA metabolism regulates several physiological mechanisms, such as glucose and lipid metabolism, immune regulation, integrity of the epithelial barrier, and host-microbiota interactions. This section discusses the available information regarding the impact of alterations in BA metabolism caused by antibiotics, starting from their effect on metabolic and immunoregulatory functions.

**TABLE 1:** Antibiotic-induced gut microbiota depletion and bile acid imbalance.

Antibiotic type	Study setting	Affected bacteria	Bile acid impacted	Result of bile acid imbalance	References
Broad-spectrum antibiotic cocktail (ampicillin, vancomycin, neomycin, and metronidazole)	Mouse model of antibiotic-induced microbiome depletion (AIMD)	Marked depletion of Firmicutes and Bacteroidetes, including bile acid (BA)-transforming bacteria possessing bile salt hydrolase (BSH) and 7α-dehydroxylation activities	Decreased production of secondary BAs such as deoxycholic acid (DCA) and lithocholic acid (LCA) (secondary BA pool collectively reduced)	Altered whole-body BA metabolism, disrupted gut signaling and GLP-1 regulation, and contributed to changes in glucose homeostasis and insulin sensitivity	Zarrinpar et al., 2018
Vancomycin	Randomized controlled trial	Significant reduction in Gram-positive bacteria, particularly Firmicutes, with a compensatory increase in Proteobacteria; reduced microbial diversity	Decreased fecal secondary BAs (reduced BA dehydroxylation) and increased postprandial plasma primary BAs	Impaired microbial conversion of primary to secondary BAs, altered BA metabolism, and reduced peripheral insulin sensitivity	Vrieze et al., 2014
Minocycline	SPF and germ-free (GF) C57BL/6 mice	Gut dysbiosis in SPF mice	Increased circulating conjugated BAs, including BAs acting as FXR antagonists; disruption of the intestinal FXR–FGF15 gut–liver signaling axis and BA homeostasis	Suppressed osteoblast function, reduced bone mass, impaired bone microarchitecture and fracture resistance through attenuated FXR signaling; impaired skeletal maturation	Carson et al., 2023
Florfenicol and azithromycin	Juvenile C57BL/6 mice	Reduced gut microbial richness and diversity; increased Firmicutes/Bacteroidetes ratio; decreased *Christensenella*, *Gordonibacter*, *Anaerotruncus* (florfenicol), *Lactobacillus* (azithromycin), and *Alistipes*, *Desulfovibrio*, *Parasutterella*, *Rikenella* (both treatments)	Decreased colonic secondary BAs and increased primary BAs	Impaired microbial BA transformation, altered BA metabolism, increased adipogenesis and body fat accumulation, suggesting a potential role of antibiotic-induced dysbiosis in childhood obesity	Li R. et al., 2017
Rifampicin	HepG2 cell culture model	No microbiota evaluated	Increased extracellular total BAs (TBA) and altered BA transport through modulation of BSEP, MDR1, MRP2, NTCP, OATP2, and OSTβ transporters	BA accumulation, oxidative stress, ER stress, hepatocellular injury, and apoptosis; activation of Nrf2-mediated adaptive response partially protected against injury	Zhang et al., 2017
Cefoperazone	Wild-type (WT) and MR1−/− (MAIT-cell-deficient) mice	Cefoperazone depleted gut bacteria involved in BA metabolism; WT mice exhibited greater microbiota disruption than MR1−/− mice	Increase in taurocholic acid (TCA) and decrease in deoxycholic acid (DCA) in WT mice	Loss of secondary BA production and accumulation of primary BAs; BA profile associated with increased susceptibility to *Clostridium difficile* colonization in WT mice, whereas MR1−/− mice maintained higher DCA levels and showed resistance to colonization	Sun et al., 2020
Antibiotic cocktail (ABX) (ampicillin, neomycin sulfate, metronidazole, and vancomycin)	Experimental autoimmune prostatitis (EAP) mouse model	Antibiotic treatment significantly altered gut microbial composition; Fecal microbiota transplantation (FMT) from ABX-treated mice transferred the phenotype	Decreased deoxycholic acid (DCA)	Reduced DCA relieved its suppressive effect on FXR, leading to enhanced FXR signaling, inhibition of the NLRP3–IL17A pathway, reduced Th17 differentiation, and attenuation of autoimmune prostatitis	Shao-Yu et al., 2025
Vancomycin	Inulin-fed Toll-like receptor 5 knockout (T5KO) mice	Depletion of Lachnospiraceae, Ruminococcaceae (Firmicutes), Bifidobacteria (Actinobacteria), and *Clostridium* cluster XIVa (secondary BA producers)	Marked reduction in circulating secondary BAs; increased proportion of hydrophilic primary BAs and higher conjugated/unconjugated BA ratio	Prevention of microbiota-dependent liver cancer despite persistent cholestatic liver injury; findings suggest secondary BAs contribute to liver tumorigenesis in dysbiotic mice	Singh et al., 2020
Moxifloxacin, ceftazidime, ceftriaxone, piperacillin-tazobactam, cefoperazone-sulbactam, latamoxef, minocycline, and mezlocillin-sulbactam	Advanced NSCLC patients and Lewis lung carcinoma (LLC) mouse model	Increased Proteobacteria, Cyanobacteria, and *Deinococcus*; decreased Firmicutes, Bacteroidetes, and Verrucomicrobia	Decreased DCA levels in fecal and serum samples	Reduced efficacy of chemoimmunotherapy, shorter overall survival and progression-free survival, impaired antitumor immune responses; DCA supplementation restored antitumor effects and inhibited tumor growth	Xu et al., 2026
Gentamicin and amikacin	Colorectal cancer (CRC) liver metastasis mouse model	Reduced abundance of intestinal bacteria capable of 7α-dehydroxylation (conversion of cholic acid (CA) to DCA) and altered overall gut microbial diversity	Decreased DCA in feces and liver tissues	Inhibition of CRC liver metastasis; reduced production of the tumor-promoting secondary BA DCA	Deng et al., 2022
Penicillin, streptomycin, vancomycin, levofloxacin, clindamycin	Multiple murine tumor models receiving immune checkpoint therapy (ICT)	Antibiotic-induced depletion of microbiota responsible for producing immunomodulatory BA metabolites	Decreased taurodeoxycholic acid (TDCA)	Reduced responsiveness to ICT; impaired CD8^+^ T-cell-mediated antitumor immunity; TDCA supplementation restored ICT efficacy through TGR5 signaling	Li et al., 2026
Gentamicin and vancomycin	Pediatric intestinal failure patients with cholestasis and antibiotic-treated mouse models	Gentamicin reduced *Eubacterium* and *Bacteroides*; vancomycin reduced *Clostridium*, *Bifidobacterium*, and *Lactobacillus*; overall reduction in microbial diversity and BA-biotransforming bacteria	↓ Secondary BAs in colonic contents; ↑ primary BAs in plasma; altered tauro-β-muricholic acid (TβMCA) levels	Inhibition of intestinal FXR signaling, ↓ FGF19, ↑ hepatic CYP7A1 expression, disrupted BA synthesis and transport, leading to cholestasis and intestinal failure-associated liver disease (IFALD)	Xiao et al., 2018
Cefoperazone, clindamycin, and vancomycin	Mouse model evaluating antibiotic-induced susceptibility to *C. difficile* colonization	Significant loss of Lachnospiraceae and Ruminococcaceae, key secondary BA-producing bacteria	Marked reduction in secondary bile acids including DCA, lithocholate (LCA), ursodeoxycholate (UDCA), hyodeoxycholate (HDCA), and ω-muricholate	Loss of colonization resistance and increased susceptibility to *C. difficile* infection (CDI); reduced secondary BAs allowed spore germination and vegetative outgrowth	Theriot et al., 2016
Vancomycin and ridinilazole	Longitudinal clinical study of patients treated for CDI	Vancomycin depleted Bacteroidales and Clostridiales families and promoted Enterobacteriaceae expansion; ridinilazole largely preserved these microbiota	Vancomycin caused a nearly 100-fold increase in the conjugated-to-secondary BA ratio; marked loss of secondary BAs and accumulation of conjugated primary BAs. Ridinilazole maintained BA profiles near baseline	Vancomycin-induced BA dysbiosis was associated with increased risk of CDI recurrence, whereas preservation of microbiota-dependent BA metabolism by ridinilazole was associated with lower recurrence risk	Qian et al., 2020
Cefoperazone	Mouse model of antibiotic-induced susceptibility to *Candida albicans* gastrointestinal colonization	Cefoperazone-induced gut dysbiosis (specific taxa not detailed in abstract); loss of microbiota-associated BA metabolism	↑ Primary BAs and ↓ secondary BAs in the cecum	Altered intestinal metabolic environment promoted *C. albicans* growth, morphogenesis, and GI colonization; reduced secondary BAs removed inhibitory effects against fungal expansion	Gutierrez et al., 2020
Ampicillin, vancomycin, metronidazole, and neomycin	Mouse liver ischemia–reperfusion injury (IRI) model	Antibiotic-induced microbiota remodeling; decreased Firmicutes and increased *Clostridium* species involved in BA transformation	Increased unconjugated BAs, particularly UDCA, which act as FXR agonists	Activation of FXR reduced TLR4 expression, inhibited MAPK/NF-κB inflammatory pathways, decreased CCR2^+^ monocyte recruitment, apoptosis, and liver injury	Liu et al., 2022b
Vancomycin	Experimental autoimmune encephalomyelitis (EAE) mouse model	Enrichment of 6 taxa within the Clostridia vadin BB60 group after vancomycin treatment	Decreased DCA	Reduced DCA together with increased indole-3-lactic acid altered astrocyte mTOR pathway gene expression and was associated with improved EAE recovery	Bianchimano et al., 2025
Vancomycin	Tsumura-Suzuki non-obese mice fed a high-fat/cholesterol/cholate (iHFC) diet as a model of advanced NASH and liver fibrosis	Depletion of Gram-positive gut bacteria; vancomycin produced greater microbiota alteration than metronidazole	Altered BA levels and composition	Promoted progression of liver damage, steatohepatitis, and fibrosis; increased hepatic macrophage recruitment (F4/80^+^ and CD11c^+^ macrophages) and crown-like structures, indicating that antibiotic-induced microbiota and BA changes contribute to NASH progression	Kasai et al., 2023
Gentamicin	Rat model of 17α-ethynylestradiol (EE)-induced cholestatic liver injury and healthy rats;	Reduced abundance of *Lactobacillus* and *Bacteroides*, both important BSH-expressing bacteria	↓ Total BA in serum and liver; ↑ ratio of primary/secondary BAs and ↑ ratio of conjugated/unconjugated BAs	Reduced microbial BSH activity, leading to accumulation of hydrophilic conjugated BAs, increased urinary BA excretion, decreased hepatic BA accumulation, and alleviation of cholestatic liver injury.	Ma et al., 2023
Vancomycin and amoxicillin	Single-blinded randomized controlled trial	Vancomycin reduced microbial diversity with depletion of Gram-positive bacteria, mainly Firmicutes, and increased Gram-negative bacteria, mainly Proteobacteria; amoxicillin showed no significant effects	↓ Secondary BAs in feces and ↑ primary BAs in plasma; reduced microbial BA dehydroxylation activity	Disruption of BA metabolism and reduced peripheral insulin sensitivity; changes in fecal BAs were strongly associated with altered Firmicutes abundance	Vrieze et al., 2014

### 4.1 Metabolic homeostasis

Recently, the influence of gut microbiota on enhancing insulin sensitivity has been shown to be mediated via an FXR-induced increase in the FGF19 signaling pathway. In a research, administration of vancomycin was found to induce profound reductions in Gram-positive bacteria, especially those belonging to the Firmicutes group, specifically from *Clostridium* clusters IV and XIVa (Vrieze et al., 2014). These changes were accompanied by a significant reduction of secondary BAs found in the feces. The deficiency in secondary BAs might have caused a drop in FGF19 concentrations, which correlated with enhanced BA production in the liver as demonstrated by increased plasma C4 concentrations. BAs have recently been acknowledged not only as factors promoting lipid uptake by the intestine, but as important regulators of human metabolism. BA receptors and subsequent signaling pathways regulate various metabolic processes such as energy balance, gluconeogenesis, insulin secretion, and insulin sensitivity (Vrieze et al., 2014). Therefore, BA modulation can become an important target for combating insulin resistance in obese individuals. In the Vrieze et al. (2014) work, oral vancomycin administration has caused a significant decrease in secondary BAs in the feces that might explain the impaired peripheral insulin sensitivity. It has been shown that some bacteria responsible for the production of short-chain fatty acids, such as *Faecalibacterium prausnitzii* and *Eubacterium hallii*, have been connected to secondary BA metabolism. Consequently, there is a possibility of a mutual relationship between BA profiles, short-chain fatty acids (SCFAs)-producing microbes, and FXR regulation of glucose metabolism (Vrieze et al., 2014).

Kuno et al. (2018) proved that the short-term exposure of conventional mice to antibiotics (5 days) induced a decline in serum glucose, serum triglycerides (TG), liver TG concentration, and liver weight. The effects were reversible with the addition of low-dose secondary BAs because the secondary BAs in the liver recovered to their baseline levels. The data prove that secondary BAs synthesized by gut microbiota play a crucial part in metabolic regulation. The effect is in agreement with the previously reported findings that norfloxacin and ampicillin-induced antibiotic exposure in 2 weeks resulted in decreased blood glucose levels and hepatic triglyceride concentration in obese ob/ob mice (Membrez et al., 2008).

The activation of two types of nuclear receptors, which respond to BAs, PXR and VDR, causes plasma TG levels to increase (Fiorucci et al., 2010). It should be noted that the reduced levels of secondary BAs and lowered serum TG levels were induced by antibiotic exposure. Since LCA displays higher agonistic effects toward PXR and VDR than all other BAs, the reduction in secondary BAs could be expected to inhibit the PXR and VDR signaling, resulting in the decrease in circulating TG levels (Staudinger et al., 2001; Makishima et al., 2002). Despite this fact, the TG levels remained unchanged after antibiotic exposure.

Antibiotic therapy (vancomycin and polymyxin B) caused a significant reduction in the levels of TLCA and TDCA (Kuno et al., 2018). Under these conditions, the increase in BA biosynthesis was expected due to a weaker feedback inhibition on cholesterol/BA pathways, confirmed by the increase in protein expression involved in the processes, which normalized after secondary BA supplementation. Corresponding to this, increased concentrations of the primary BAs (Taurochenodeoxycholic acid (TCDCA), taurocholic acid (TCA), and TβMCA) were observed. Particularly, the action of TCDCA (and CDCA) on FXR was found relatively strong compared to LCA and DCA55, while TβMCA is an FXR inhibitor, implying that antibiotic-induced changes in BA composition had a significant effect on FXR activity (Kuno et al., 2018). The FXR activation is responsible for lowering the blood glucose and TG levels, promoting liver regeneration, and regulating metabolic enzymes (Cyp7a1 and Cyp51a1 inhibition and Cyp3A4/Cyp3a11 induction) (Gnerre et al., 2004; Huang et al., 2006). Consequently, decreased liver weight, changed the expression of enzymes synthesizing BAs (increase in Cyp7a1 and Cyp51a1 levels), and decreased Cyp3a11 were, at least partially, mediated by the FXR inhibition caused by high TβMCA concentrations. On the other hand, lowered blood glucose and TG levels might have been caused by the FXR agonism triggered by TCDCA. Overall, various FXR and non-FXR nuclear receptor signaling pathways appear to interact with each other to elicit metabolic effects of antibiotic-induced changes in secondary BAs. Proteomics analysis also confirmed this effect by showing numerous protein changes in the liver associated with carbohydrate and lipid metabolism under experimental conditions.

Antibiotic-induced microbiome depletion (AIMD) results in significant changes in secondary BAs in the intestinal lumen, which have an effect on metabolic pathways in host cells through modulation of intestinal luminal signals (Zarrinpar et al., 2018). Specifically, AIMD is associated with decreased levels of circulating glucose and insulin sensitivity and increased insulin secretion and gluconeogenesis, which takes place together with upregulated levels of GLP-1 (Zarrinpar et al., 2018). Such effects are thought to be linked with elevated levels of primary BAs, especially TCA, in the intestinal lumen. Besides the influence of AIMD on glucose utilization in the intestine, AIMD also contributes to a selective increase in BA uptake from the intestine. Moreover, AIMD has a strong impact on BA physiology, and although FXR agonists and antagonists (TCA and TβMCA, respectively) are both elevated in the intestinal pool of BAs, the expression of FXR itself and its targets (including Ostβ and Ibabp) is increased in the cecum, which reflects enhanced intestinal FXR signaling. It can be suggested that such changes are responsible for enhanced BA absorption, which is proved by the fact that serum pools of BAs become more similar in AIMD and control mice. At the same time, the intestinal absorption seems to be selective for TβMCA, and the decrease of its concentration in the liver leads to reduced FXR activity. Such an effect is confirmed by the decrease in expression of Shp, a target of the FXR gene, as well as the upregulation of Cyp7a1-dependent synthesis of BAs (Zarrinpar et al., 2018). It is unlikely that the difference in TGR5 expression in AIMD-treated and control mice explains all the differences in BA effects on glucose metabolism; however, AIMD-associated shifts in BA pool composition can influence TGR5-dependent signaling, which, in turn, contributes to glucose utilization. Finally, AIMD increases the level of another BA, tauroursodeoxycholic acid (TUDCA), which is known for its hepatoprotective and anti-inflammatory properties, which might reduce inflammation (Llopis et al., 2016).

### 4.2 Immunomodulation

Findings obtained in the present research and those of previous investigations suggest that antibiotic-driven shifts in BA metabolism of the gut microbiota are critical for the regulation of *C. difficile* germination and colonization (Theriot et al., 2014; Kochan et al., 2017). Additionally, the transformation of BAs by microbes (e.g., deconjugation and oxidation of hydroxyl groups) is carried out by the gut microbiota (Ridlon et al., 2006; Swann et al., 2011). On the other hand, BAs are important for the development of gut immunity since they control the activities of immune cells and modulate the balance of T cells producing Interleukin (IL)-17a and regulatory T cells (Hang et al., 2019). Nevertheless, effects of antibiotics on BA metabolism in mice without MAIT cells have not been previously investigated. Thus, Sun et al. (2020) aimed to compare BA profiles and their responses to antibiotic administration in knockout (KO) and wild-type (WT) mice. Earlier research showed that treatment of mice with cefoperazone through water feeding significantly impaired their gut microbiota and resulted in about three orders of magnitude fewer microorganisms as well as changes in microbial population structure (Antonopoulos et al., 2009). Similarly to previous findings, a previous study confirmed the lower sensitivity of the gut microbiota of *MR1−/−* mice to cefoperazone treatment relative to that of WT animals (Smith et al., 2019). To evaluate whether antibiotic-driven shifts in BA metabolism are similarly affected, total intensity of BAs in cecum content and feces was assessed in KO and WT mice. Interestingly, KO mice had greater total intensity of BAs in comparison with WT mice regardless of antibiotic treatment. Changes in abundance and distribution of BAs in cefoperazone-treated KO and WT mice were significantly different from each other as observed for the gut microbiota in THE previous study (Smith et al., 2019).

Cefoperazone administration resulted in elevated concentrations of TCA, a taurine-conjugated primary BA, in cecal contents and feces of WT mice (Sun et al., 2020). It can be explained by the decreased activity of bacteria associated with the reduction in the numbers of microorganisms due to antibiotic treatment. It was established that cefoperazone resulted in lower abundance of Lactobacillaceae in the gut of WT mice (Smith et al., 2019). Reduction in free taurine concentration in the feces of WT mice indicates an increase in TCA abundance due to taurine binding with cholic acid. DCA, a secondary BA, was less abundant in the feces of both KO and WT mice treated with cefoperazone. This may be attributed to the lower activity of bacterial populations responsible for performing 7α-dehydroxylation reactions. Earlier studies have found the depletion of Clostridiales (including *Clostridium scindens*, an important bacterium in secondary BA synthesis) in cefoperazone-treated WT mice (Ridlon et al., 2006).

It has already been reported that the activity of Th17 cells is regulated by the composition of gut BAs (Hang et al., 2019). Changes in the composition of the microbiome and BAs were shown to affect Th17 immune reactions, especially in inflammatory bowel disease (IBD; Yan et al., 2023). It was first suggested that changes in the composition of the microbiome and its metabolite production should occur for the complete action of antibiotics to influence both T helper (Th) 17 cell differentiation and the signaling of BAs (Shao-Yu et al., 2025). Indeed, in accordance with this assumption, the use of Fecal microbiota transplant (FMT) based on microbiota from antibiotic-treated mice resulted in a significant decrease in inflammatory infiltration in the prostate in the experimental model of autoimmune prostatitis (EAP; Shao-Yu et al., 2025). This was associated with improved gut microbial balance and BA metabolism. Altogether, the obtained results imply that the post-antibiotic state of the microbiome is necessary and could be sufficient for achieving the beneficial effect of antibiotics in terms of FMT (Shao-Yu et al., 2025). As mentioned above, the level of Th17 cell differentiation directly determines the extent of immune cell infiltration and the degree of prostate inflammation (Stellwag and Hylemon, 1979; Motrich et al., 2020). The use of microbiota from antibiotic-treated donors in the current work revealed an important feature of antibiotic-shifted microorganisms – the ability of the latter to inhibit Th17 differentiation in recipient mice. Therefore, the presence of these properties indicates that the microorganism can be used as a base for designing new microbiome-based methods for the treatment of chronic pelvic pain syndrome/Chronic prostatitis (CPPS/CP).

**FIGURE 2:**
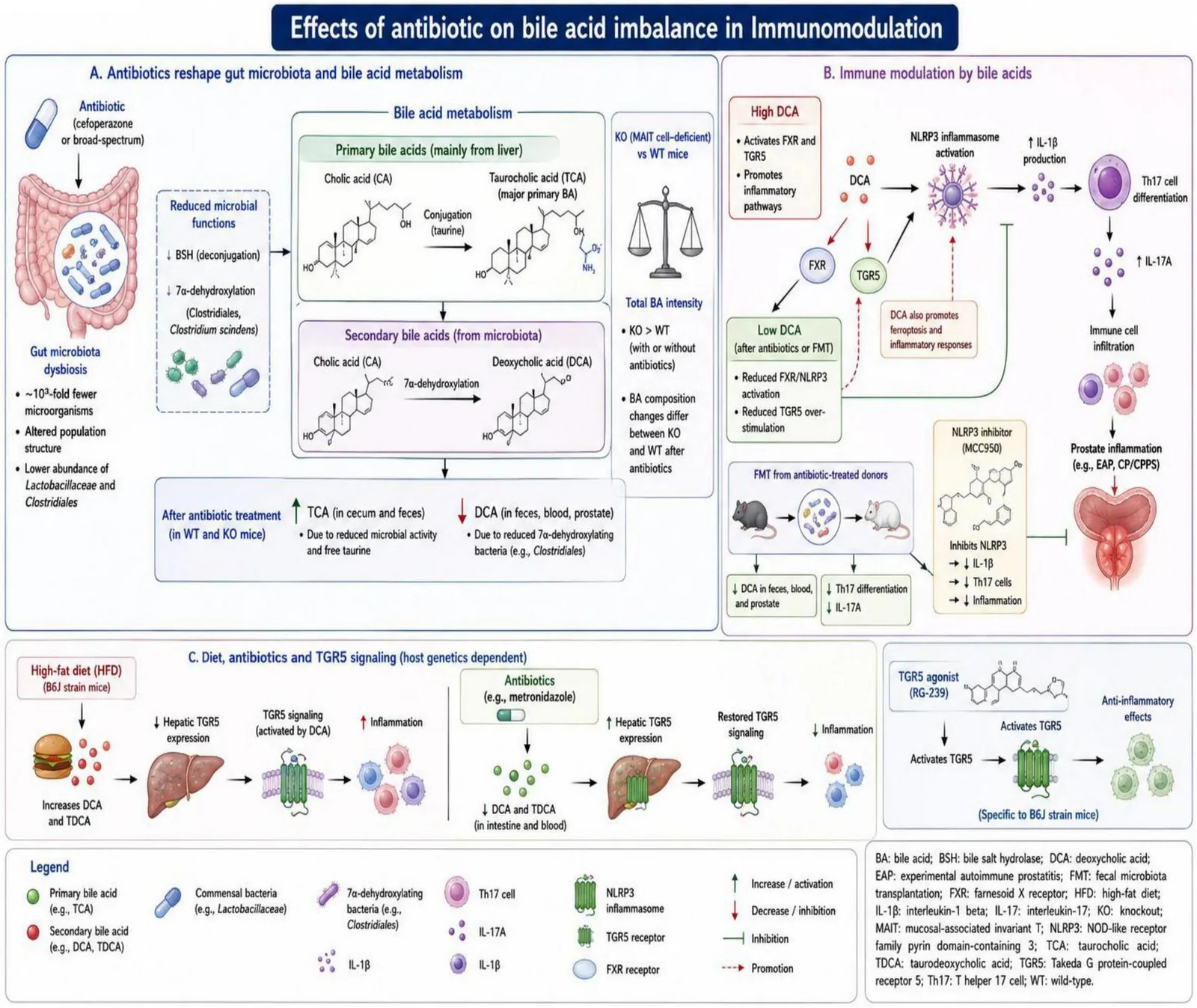
Immunomodulatory effects of gut microbiota-derived bile acids and antibiotic-induced alterations in bile acid metabolism. Antibiotic treatment alters the composition and metabolic activity of the gut microbiota, leading to profound changes in bile acid (BA) metabolism and host immune responses. Broad-spectrum antibiotics such as cefoperazone reduce populations of bile salt hydrolase (BSH)- and 7α-dehydroxylation-producing bacteria, including members of the Lactobacillaceae and Clostridiales families, resulting in the accumulation of taurine-conjugated primary BAs, particularly taurocholic acid (TCA), and a reduction in secondary BAs such as deoxycholic acid (DCA). These antibiotic-induced alterations in BA composition influence immune cell differentiation and inflammatory signaling pathways. Changes in gut microbial communities and BA profiles regulate the balance between pro-inflammatory T helper 17 (Th17) cells and regulatory immune responses. Fecal microbiota transplantation (FMT) from antibiotic-treated donors suppresses Th17-cell differentiation, reduces inflammatory cell infiltration, and alleviates inflammation in experimental autoimmune prostatitis (EAP). Reduced production of DCA following antibiotic treatment or Fecal microbiota transplantation (FMT) is associated with decreased activation of the FXR–NLRP3 signaling axis, suppression of IL-1β production, and inhibition of Th17-cell differentiation. DCA serves as a key microbiota-derived immunomodulatory metabolite that promotes inflammatory responses and may induce ferroptosis-related signaling. Alterations in DCA metabolism affect multiple pathways involved in cholesterol biosynthesis, BA-responsive transcriptional networks, FXR signaling, and NLRP3 inflammasome activation. Pharmacological inhibition of NLRP3 using MCC950 attenuates inflammation and reduces Th17-cell responses, supporting the role of the DCA–NLRP3–Th17 axis in immune regulation. In obesity-associated inflammation, high-fat diet (HFD)-induced increases in DCA and its conjugates contribute to inflammatory responses through BA receptor signaling. Antibiotic treatment reduces intestinal and circulating levels of DCA and taurodeoxycholic acid (TDCA), restores hepatic expression of the G protein-coupled BA receptor TGR5, and exerts anti-inflammatory effects.

Shao-Yu et al. (2025) further revealed the role of BA metabolism in the above-discussed events. As was shown before, FMT from antibiotic-treated EAP animals to recipients resulted in the reduction of the levels of DCA in feces, blood, and prostate gland. Thus, these results, in addition to metabolomic and microbiome analysis, indicate the involvement of bacterial activity in the control of immune reaction through the regulation of DCA metabolism (Shao-Yu et al., 2025). Consistent with this, it was found that there are some bacterial species that are directly connected with inflammatory response, e.g., *Bacteroides* are associated with an anti-inflammatory effect, while Alistipes are more common for dysbiosis and inflammation (Parker et al., 2020; Price et al., 2024). It was recently discovered that DCA promotes an inflammatory response by stimulating ferroptosis (Li et al., 2023). Thus, it can be suggested that the interaction between bacteria and host metabolism and immune response involves DCA metabolism as the central component. Changes in levels of DCA-related metabolites may affect signaling pathways including those controlling cholesterol biosynthesis, BA-responsive transcriptional network, and FXR receptor activity. Shao-Yu et al. (2025) showed that FMT after antibiotic treatment influences the inflammatory infiltration of the prostate by FXR–NLRP3 pathways. It was confirmed by proteomic analyses that these alterations are associated with the activation of Th17 cell differentiation and IL-17 signaling pathways as well as the regulation of cholesterol metabolism. Moreover, in agreement with earlier reports, it was established that the NLRP3 inflammasome plays a crucial role in Th17 cell differentiation (Figure 2) (Bai et al., 2022; Liu et al., 2024). NLRP3 activation occurs in the case of EAP, and pharmacological inhibition of the NLRP3 inflammasome using MCC950 results in a reduction of the inflammation associated with increased DCA levels as well as in suppression of Th17 differentiation. Indeed, MCC950 inhibits NLRP3, which leads to the inhibition of IL-1β synthesis and, as a result, a reduced number of Th17 cells (Hu et al., 2024). The cellular effects of BA signaling in autoimmune prostatitis are likely mediated through coordinated interactions between intestinal epithelial cells and immune cell populations rather than through a single cellular target. FXR is expressed in intestinal epithelial cells, macrophages, dendritic cells, and subsets of T lymphocytes, whereas TGR5 is predominantly expressed on macrophages, dendritic cells, monocytes, and other myeloid cells. In the context of antibiotic-induced alterations in DCA metabolism, reduced activation of these BA-responsive pathways is thought to modulate macrophage and antigen-presenting cell function, thereby regulating NLRP3 inflammasome activation and IL-1β production. Because IL-1β is a key cytokine driving Th17-cell differentiation, attenuation of the FXR–NLRP3 axis ultimately limits Th17 polarization and inflammatory cell infiltration. Simultaneously, BA receptor signaling in intestinal epithelial cells contributes to maintenance of epithelial barrier integrity, thereby reducing microbial translocation and systemic inflammatory signals that further amplify autoimmune responses. Together, these findings support a model in which microbiota-derived BAs coordinate both epithelial and immune cell functions to regulate inflammation in autoimmune prostatitis, although the relative contribution of individual cell populations remains an active area of investigation. Overall, under a standard chow diet, BA profiles in the plasma revealed that about 2.6%–5.5% of BAs in circulation were present as conjugates of DCA. In contrast, high-fat diet (HFD) feeding was associated with the increased presence of pro-inflammatory BA, DCA, only in the B6J strain mice, while this HFD-induced effect was abrogated by the use of antibiotics (Fujisaka et al., 2016). As a result, Fujisaka et al. (2016) hypothesized that the contribution of one of the most important components of the anti-inflammatory effect of antibiotics on B6J mice is due to the inhibition of gut microbes capable of transforming primary BAs into DCA and its conjugates. In the current study, the role of BAs in inflammatory reactions in B6J mice seems to involve BA-induced signaling via the TGR5 receptor, yet this process could be modulated by host genetics as well. In this regard, they found that the expression of TGR5 protein in the liver was significantly downregulated by the administration of HFD. On the other hand, the application of broad-spectrum antibiotics, especially metronidazole, resulted in a recovery of TGR5 levels, which was accompanied by a decrease in the concentrations of secondary BAs, namely, DCA and TDCA, in the intestines and blood serum (Fujisaka et al., 2016). Since DCA is considered a potent activator of the TGR5 receptor, this finding suggests the participation of DCA and other secondary BAs in the restoration of hepatic TGR5 levels. Stimulation of TGR5 by its selective agonist, RG-239, demonstrated anti-inflammatory effects; however, these effects were specific for the B6J strain only since the TGR5 expression levels did not differ between the three strains analyzed (Fujisaka et al., 2016).

## 5 Antibiotic and bile acid metabolism in terms of diseases

Antibiotic exposure disrupts the composition and functional capacity of the gut microbiota by selectively depleting bacterial taxa responsible for BA biotransformation, particularly BSH-producing microorganisms and 7α-dehydroxylating bacteria. As a consequence, the conversion of conjugated primary BAs into unconjugated and secondary BAs is markedly reduced, resulting in an altered bile acid pool characterized by the accumulation of conjugated primary BAs and depletion of bioactive secondary BAs, including DCA and LCA. Because individual BA species exhibit distinct affinities for host receptors, these compositional changes profoundly influence BA-dependent signaling networks that coordinate metabolism, immunity, and tissue homeostasis. Reduced activation of FXR disrupts the physiological feedback regulation of BA synthesis and transport, impairs lipid and glucose metabolism, and compromises intestinal barrier integrity. Simultaneously, diminished stimulation of TGR5 decreases GLP-1 secretion, energy expenditure, and anti-inflammatory signaling while altering macrophage polarization and innate immune responses. Loss of secondary BA also promotes activation of the NLRP3 inflammasome, leading to excessive production of pro-inflammatory cytokines, including IL-1β and IL-18, thereby amplifying intestinal and systemic inflammation. In parallel, dysregulated BA signaling influences mTOR activity, affecting cellular metabolism, autophagy, immune-cell differentiation, and tissue repair. Collectively, these interconnected signaling pathways establish a mechanistic link between antibiotic-induced microbial dysbiosis and the development of metabolic disorders, infectious diseases, liver injury, neurological disorders, and cancer.

It is critical to note that the biological impact of BAs, including DCA, is highly dependent on context and is not necessarily good or bad. Under normal physiological conditions, DCA acts as an essential microbial metabolite and a factor in the maintenance of intestinal homeostasis, as it activates BA receptors, including TGR5 and less FXR, and regulates immune response, epithelial barrier function, energy metabolism, and colonization resistance against enteric pathogens. On the other hand, a prolonged effect of high DCA concentrations, especially in the case of obesity, high-fat diet, dysbiosis, or disturbed BA homeostasis, leads to cytotoxicity due to oxidative stress, DNA damage, mitochondrial dysfunction, cell senescence, and inflammation. Thus, depending on various parameters, including the level of DCA, the period of exposure, the presence of tissue-specific receptors, the microenvironment, and pathology, the biological consequences of DCA signaling are different. Such context dependency makes possible the positive role of DCA in intestinal homeostasis and protection from infections but also in tumorigenesis at the same time.

This integrated framework highlights that the pathological consequences of antibiotics are not solely attributable to microbial depletion but also arise from disruption of microbiota-derived BA metabolism and the downstream signaling pathways that maintain host physiological homeostasis. Antibiotics alter the structure and function of the intestinal BA pool by disrupting the gut microbiota, which impacts many diseases. Antibiotic-induced BA dysbiosis can have adverse effects not only on the physiology of the gut but also on other organs and diseases. Recent studies have shown that alterations in microbial BA metabolism can affect susceptibility to infectious, metabolic, hepatic, neurodegenerative diseases, and cancer. The next sections provide an overview of the available mechanistic data on the role of antibiotic-induced BA alterations in the disease states.

### 5.1 Antibiotic and bile acid metabolism in infection

**FIGURE 3:**
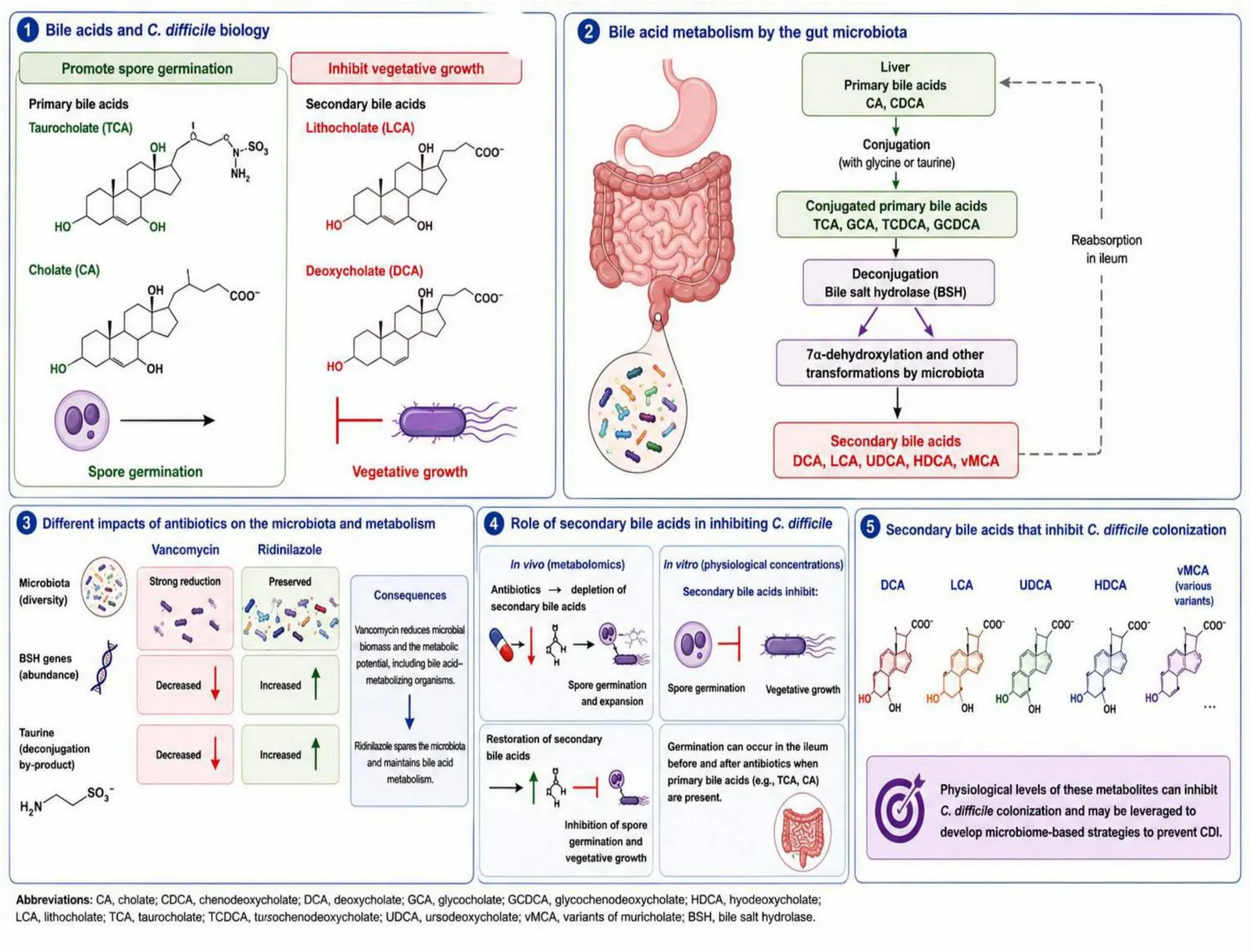
Antibiotic and bile acid metabolism during infection. (1) Bile acids (BAs) and *Clostridium difficile* biology. Primary BAs, including taurocholate (TCA) and cholate (CA), act as germinants that promote the transition of dormant *C. difficile* spores into vegetative cells. In contrast, secondary BAs, particularly lithocholate (LCA) and deoxycholate (DCA), inhibit vegetative growth and contribute to colonization resistance against *C. difficile*. (2) Microbial metabolism of BAs. Primary BAs synthesized in the liver [e.g., cholate (CA) and chenodeoxycholate (CDCA)] are conjugated with taurine or glycine before secretion into the intestine. Gut microbiota expressing bile salt hydrolase (BSH) enzymes deconjugate these BAs, followed by 7α-dehydroxylation and other microbial transformations that generate secondary BAs, including DCA, LCA, ursodeoxycholate (UDCA), hyodeoxycholate (HDCA), and muricholate variants (vMCA). A portion of BAs is reabsorbed in the ileum and recycled through enterohepatic circulation. (3) Differential effects of antibiotics on gut microbiota and BA metabolism. Broad-spectrum antibiotics such as vancomycin markedly reduce microbial diversity and biomass, leading to decreased abundance of BSH-producing bacteria and reduced BA-transforming capacity. Consequently, production of secondary BAs declines. In contrast, ridinilazole preserves a greater proportion of the indigenous microbiota, maintaining BSH activity, taurine release during deconjugation, and secondary BA production. These differences influence susceptibility to *C. difficile* colonization and recurrence. (4) Role of secondary BAs in suppressing *C. difficile*. Antibiotic-induced depletion of secondary BAS removes an important barrier to *C. difficile* infection (CDI), allowing spore germination, vegetative growth, and intestinal expansion. Restoration of secondary BA pools re-establishes colonization resistance by inhibiting both spore germination and vegetative cell proliferation. Although germination can occur in the ileum where primary BAs are present, secondary BAs in the lower intestine restrict pathogen expansion. (5) Secondary BAs associated with inhibition of *C. difficile* colonization. Several physiologically relevant secondary BAs, including DCA, LCA, UDCA, HDCA, and vMCA, exhibit inhibitory activity against *C. difficile*. These metabolites represent important microbiota-derived factors contributing to colonization resistance and may serve as targets for microbiome-based therapeutic strategies aimed at preventing or reducing *C. difficile* infection (CDI).

A growing number of findings show the correlation between the BAs’ spectrum and the development of diseases and their recurrence (Shen, 2015; Winston and Theriot, 2016). One of the main risk factors that predisposes to *C. difficile* infection (CDI) is a disturbance of the gut microbiota as a consequence of antibiotics (Figure 3). *In vitro* studies have revealed that *C. difficile* spore germination was promoted by primary BAs, namely, conjugated TCA and unconjugated CA, while vegetative cell growth was inhibited by secondary BAs, LCA and DCA. In Qian et al. (2020) study, it was found that ridinilazole and vancomycin affected the composition of BAs in fecal samples of patients with CDI differently at the end of treatment (EOT) and in the post-therapy period. As opposed to baseline, EOT showed an increase in primary and conjugated BAs together with a strong reduction in secondary BAs in the case of vancomycin use.

The difference was also revealed in the changes of BAs’ profile induced by ridinilazole. In particular, the composition of the BAs was preserved in the case of ridinilazole compared with baseline and even approached the spectrum of the healthy population 40 days after the diagnosis of CDI was made (Qian et al., 2020). The results obtained could be due to different effects on the microbiota caused by these two drugs, because secondary BAs are produced as a result of the transformation of conjugated BAs through enzymes expressed by some species of the microbiota in the small intestine and colon (Wahlström et al., 2016). Indeed, in Qian et al. (2020), a higher abundance of BSH genes and an elevation of taurine concentration (the by-product of deconjugation) were detected. Earlier research has proven that vancomycin leads to a reduction in the biomass of bacteria, which, besides influencing microbial diversity, also results in a decrease in the metabolic potential of microbiota, including BA-metabolizing organisms (Thorpe et al., 2018). In addition to targeting the intestinal microbiota, Theriot et al. (2016) investigated the ability of *in vivo* secondary BA concentrations, which they measured using metabolomics, to inhibit spore germination and expansion in the small and large intestine. *C. difficile* spores were shown to be able to germinate and expand when the amount of secondary BAs was depleted, and the gut microbiota suffered extensive damage due to antibiotic treatment. In order to reveal colonization resistance-related inhibition, Theriot et al. (2016) performed *in vitro* inhibition experiments and demonstrated that physiologically relevant concentrations of secondary BAs can inhibit both spore germination and vegetative growth in the large intestine. Interestingly, they revealed the capability of *C. difficile* spores to germinate in the ileum both before and after antibiotics. Consistent with several earlier studies conducted in rodents, the results indicated germination of the spores in the small intestine (Wilson et al., 1985; Koenigsknecht et al., 2015). While primary BAs are normally absorbed in the small intestine, there must still be enough of them in order to initiate spore germination. Indeed, the presence of germinant molecules such as TCA and CA was detected in the ileal content under both non-antibiotic and antibiotic conditions, as reported previously (Sayin et al., 2013). Although the enhancement of DCA levels in the gut has been a major focus in many research studies, higher DCA levels have been found to pose a risk of colorectal cancer (CRC; Bernstein et al., 2005; Mcgarr et al., 2005). In this respect, the Theriot et al. (2016) study revealed that the metabolome contained additional secondary BAs, including LCA, UDCA, hyodeoxycholate (HDCA), as well as various variants of muricholate (vMCA), which were able to inhibit *C. difficile* colonization at physiological concentration levels. These metabolites can prove helpful in developing strategies aimed at preventing colonization with *C. difficile* through the modulation of the gut microbiome. Taken together, these findings show that depletion of BAs caused by antibiotics is the main mechanism responsible for the loss of colonization resistance against *C. difficile*. Disruption of BA metabolism due to a reduction in microbial BA metabolism promotes germination and proliferation of the bacteria while limiting the protective properties of secondary BAs like DCA and LCA.

### 5.2 Neurological diseases

There is increasing recognition that antibiotics can induce changes in BA metabolism of microorganisms, thus affecting the interaction between gut and brain and leading to the development of neurological diseases. While BAs control the physiology of the intestine, they can also serve as signaling molecules and affect neuroinflammation, glial functions, cell differentiation, and neuronal metabolism (Butcher and Arthur, 2025; Pei et al., 2026). In recent years, increasing evidence from animal and clinical studies supports the involvement of gut microbiota in different neurological disorders. Bianchimano et al. (2025) found that oral administration of vancomycin during the early stage of Experimental autoimmune encephalomyelitis (EAE) significantly promoted disease recovery. Vancomycin-induced improvement in disease outcome was accompanied by an increased abundance of commensal *C. difficile* BB60 in the gut microbiome of EAE mice. They also discovered a decrease in DCA fecal concentrations in vancomycin-treated EAE mice compared with untreated and ampicillin-treated counterparts (Bianchimano et al., 2025). The role of DCA in EAE pathogenesis is unknown; however, it has been shown that DCA can exacerbate inflammatory responses in colitis models. Considering these findings and the potential roles of BAs and their secondary metabolites on inflammatory responses, it is plausible that vancomycin promotes EAE recovery via elimination of gut bacteria producing neurotoxins and pro-inflammatory BAs such as DCA and favors bacteria that produce Indole-3-lactic acid (ILA), an anti-inflammatory metabolite, in the gut lumen (Bianchimano et al., 2025). Overall fecal BA content was higher in untreated EAE mice compared with healthy mice. Specifically, the concentration of TUDCA, a neuroprotective BA, was increased in untreated EAE mice, which might be part of a compensatory mechanism that aims at alleviating neuroinflammation in EAE disease progression. On the other hand, vancomycin increased TUDCA concentration in naïve mice, while this effect was not evident in EAE animals. Given that TUDCA is known to modulate astrocyte physiology, Bianchimano et al. (2025) assessed the impact of vancomycin treatment on the expression of genes involved in astrocytic functions. Analysis of astrocyte genes revealed that vancomycin caused alterations in the expression of mTOR pathway-related genes. As reported before, inhibiting mTOR signaling improved the outcome of EAE through regulatory T cell activation and Th1/Th17 responses. The involvement of the mTOR signaling pathway in the progression of EAE via astrocytes is currently poorly understood. Recent findings demonstrated the involvement of astrocyte mTOR signaling in endolysosomal remodeling, which is a process relevant to neuroinflammation. Bianchimano et al. (2025) showed that vancomycin increased the expression of *Fdxr*, *Acsl3*, and *Nufip1* in the mTOR signaling pathway in astrocytes of EAE mice. Furthermore, increased expression of these genes negatively correlated with DCA, p-cresol, and 3-IS levels, which were higher in EAE compared with control mice. Downregulated genes after vancomycin treatment exhibited a negative correlation with ILA and a positive correlation with the levels of DCA, p-cresol, and 3-IS in EAE. Also, it should be noted that Acsl3 expression positively correlated with decreased fecal DCA levels in both *Lactobacillus reuteri*-fed and vancomycin-treated EAE mice (Bianchimano et al., 2025). This result indicates a possible effect of indole-derived metabolites and BAs in the regulation of mTOR signaling pathway gene expression in astrocytes. Although the precise mechanism linking intestinal DCA depletion to astrocytic mTOR signaling remains incompletely understood, current evidence suggests that this regulation is predominantly indirect rather than the result of direct DCA action within the central nervous system. Antibiotic-induced alterations in gut microbial metabolism reduce circulating secondary BAs while simultaneously reshaping the production of other microbiota-derived metabolites, including indole derivatives, and modifying systemic inflammatory signaling. These peripheral changes may influence astrocyte function through gut–brain axis communication involving altered cytokine profiles, BA receptor signaling (FXR and TGR5), and changes in intestinal barrier integrity that affect the systemic inflammatory milieu. In addition, neural pathways, including vagal signaling, have been proposed as complementary mediators of microbiota–brain communication, although direct evidence linking these pathways specifically to DCA-dependent regulation of astrocytic mTOR signaling remains limited. Therefore, the available data support a model in which DCA depletion contributes to astrocytic gene regulation indirectly through integrated metabolic and immune signaling rather than through direct penetration of the blood-brain barrier (BBB). Indeed, recent findings demonstrated that high levels of butyrate, an anti-inflammatory metabolite, were associated with lower DCA content. Thus, considering these findings together with Bianchimano et al. (2025) results, it is likely that indoles and BAs may act jointly to regulate gene expression in the mTOR pathway in astrocytes. Indeed, both indoles and BAs were involved in the regulation of mTOR signaling, a pathway known to play a crucial role in inflammation and neurodegeneration. In summary, the effects of antibiotics on remodeling the gut microbiota affect the structure of BAs and related metabolites, which control the process of neuroimmune communication. These changes impact the functioning of astrocytes, mTOR, and inflammation. It is possible to note the relevance of the microbiota-BA-brain axis in the etiology of neurological disorders.

### 5.3 Antibiotic and bile acid metabolism in cancer

Gut bacteria and the metabolic products of BAs have been found to be important factors in cancer development and treatment (Yao et al., 2026). The disruption of gut microbes by antibiotics causes alterations in the metabolism of BAs, which subsequently affects the immune system, tumor inflammation, and the action of immune checkpoint inhibitors (Ruiz-Patiño et al., 2020; Pei et al., 2023). Recent studies have shown that all of these interactions take place through a complex process. There is evidence of some bacterial species promoting cancer development and progression (Sears and Garrett, 2014; Chen et al., 2020). Deng et al. (2022) aimed to establish which bacteria could be responsible for CRC metastasis resistance. Using a taxonomic approach of 16S rRNA sequencing, a significant increase in abundance of *Parabacteroides goldsteinii*, *Bacteroides intestinalis*, and *Bacteroides uniformis* was found in the group of animals treated with antibiotics compared to the control group. In order to elucidate how the decreased CRC metastasis resistance was caused, immune system alteration upon antibiotic administration was evaluated. No pronounced effect on hepatic CD8^+^ T cells was detected in the group of animals receiving non-absorbable antibiotics (Deng et al., 2022). As such, it is possible to conclude that the anti-metastatic mechanism worked mainly through non-immunological channels. It has been previously shown that BAs could serve as a driver of pro-tumor signaling in primary colorectal cancer (Ocvirk and O’Keefe, 2017; Katsidzira et al., 2019). Experimental models showed that diet rich in fats, genetic predisposition to CRC, and obesity contributed to disease development through elevated BA content, intestinal barrier disruption, and IL-6/Signal transducer and activator of transcription 3 (STAT3) signaling (Wang et al., 2019). In Deng et al. (2022) study, both untargeted and targeted metabolomics found a significant difference in the content of BAs. Antibiotic administration resulted in increased primary BAs and lower content of secondary BAs due to microbiome depletion alone. Combining both fecal and hepatic samples provided by the same patients allowed to reveal DCA as the most significantly altered molecule after antibiotic intervention. DCA, as a product of microbial transformation from primary BA (CA), was reported to play a critical role in CRC and liver tumorigenesis (Bayerdörffer et al., 1993; Wang et al., 2019). Additionally, in Deng et al. (2022) study, an association between DCA reduction and CRC progression was observed due to decreased microbiome diversity and increased DCA levels. Overall, non-absorbable antibiotics significantly depleted the gut microbiome with an associated decrease in DCA content. Reduced levels of an important pro-tumor signaling factor likely led to suppressed tumorigenesis in the study.

Of note, the apparently contradictory roles of DCA in cancer are increasingly recognized as being highly context-dependent rather than mutually exclusive. Chronic elevation of DCA resulting from long-term dysbiosis, high-fat diet, impaired intestinal barrier integrity, or sustained microbial 7α-dehydroxylation promotes a pro-inflammatory and pro-tumorigenic microenvironment through epithelial injury, oxidative stress, DNA damage, and activation of oncogenic pathways. In contrast, transient or controlled modulation of DCA within an intact immune microenvironment may enhance antitumor immunity by promoting tumor antigen release, facilitating antigen presentation, and improving CD8^+^ T-cell-mediated responses during chemoimmunotherapy. These divergent effects are influenced by multiple factors, including local versus systemic DCA concentrations, intestinal barrier function, tissue-specific accumulation, receptor expression profiles (particularly FXR and TGR5), and the composition of immune cell populations within the tumor microenvironment. Therefore, interpretation of DCA function requires consideration of the biological context rather than DCA concentration alone.

Xu et al. (2026) findings lend additional weight to the body of work demonstrating that antibiotics could significantly impact the efficacy of immunotherapy against non-small-cell lung cancer (NSCLC), reinforcing the importance of incorporating the patients’ antibiotic history when devising chemoimmunotherapeutic regimens. Consistent with previous studies, the application of an antibiotic mixture resulted in marked changes in the composition of the gut microbial communities in our murine LLC subcutaneous tumor model. As predicted, the microbial diversity became notably lower in the antibiotic group due to the massive depletion of beneficial bacteria (Willmann et al., 2019). In terms of the taxonomic distribution at the phylum level, a significant decrease in the abundance of Firmicutes, Bacteroidetes, and Verrucomicrobia, as well as the corresponding increases in Proteobacteria, Cyanobacteria, and Deinococcota taxa, have been found, and these changes are consistent with previously described antibiotic-induced microbiota imbalance (Ramirez et al., 2020).

Despite having a greater number of CD4^+^/CD8^+^ T-cells infiltrating tumors, no difference in tumor size has been detected between the control and the antibiotic group during sampling (Xu et al., 2026). In the case of the PTX + anti-PD-1 combination group, a lack of change in Ki67 expression in comparison with controls was combined with a smaller number of CD4^+^/CD8^+^ infiltrations, implying that these animals may have already reached the mid-course of therapy, where tumor growth has become suppressed, yet the immune response had yet to recover. In contrast, the addition of DCA resulted in more CD4^+^ and CD8^+^ infiltrations and decreased Ki67 expression, which indicates a possible role for the gut microbiota-BA axis in the tumor immune microenvironment formation (Xu et al., 2026). Nevertheless, the administration of DCA partially recovered the immune response suppressed by antibiotics, which is evidenced by increased tumor immune infiltration and lower tumor size in DCA-treated mice (Xu et al., 2026). One should note, however, that DCA supplementation was applied only to the antibiotic-naïve animals; thus, it may indicate an alternative immune-modulatory effect rather than a rescue from antibiotic-induced immunosuppression. The differences in CD4^+^/CD8^+^ infiltration between experimental groups are likely to reflect distinct immune contexts caused by immune suppression in the case of antibiotics, and immune enhancement with BAs on the contrary, rather than inconsistencies in the experimental design (Xu et al., 2026). Among other differentially abundant metabolites, DCA stood out as the most interesting. As expected, the antibiotic application resulted in the reduced concentration of DCA, and this trend was maintained throughout chemoimmunotherapy. Furthermore, the differential abundance pattern of DCA was validated in both murine sera and human fecal samples. The secondary BA, DCA, has recently attracted considerable attention as a new immunometabolic factor affecting oncology and specifically immuno-oncology therapy outcomes (Lu et al., 2025). In particular, DCA has been shown to facilitate the release of tumor antigens and their presentation to antigen-presenting cells, enabling subsequent T-cell activation in line with many immunotherapy protocols. Thus, the ability of DCA to modulate immune activity and potentiate the presentation of tumor antigens renders this metabolite relevant as a possible co-factor in chemoimmunotherapy. Besides, DCA was found to upregulate PD-L1 expression. In agreement with previous reports, Li et al. (2026) found that antibiotic treatment significantly impairs the responsiveness of several cancer types to immunotherapy. They further demonstrated this by revealing that the immunomodulatory impacts of antibiotics are not all equal. Specifically, distinct antibiotic combinations resulted in unique microbiome disturbances, differential metabolic changes, and thus heterogeneous tumor phenotypes upon immune checkpoint therapy (ICT) treatment. Such observations provide a rationale for the disparate conclusions reached among different research groups evaluating the role of antibiotics in immunotherapy outcomes (Kulkarni et al., 2020). More specifically, Li et al. (2026) study draws attention to TDCA as a critical metabolite. In contrast to many other investigations that associate the presence of specific metabolites with immunotherapy efficacy or lack thereof, they proved that TDCA is not only capable of normalizing microbiota-related immunotherapy response but also directly boosts the functional capacity of CD8^+^ T cells. Thus, TDCA serves as a functionally defined mediator of microbiota-dependent immunotherapy response.

Several studies have started unraveling the mechanism through which BAs signal to mediate antitumor immunity, though these effects are highly context-dependent. For example, DCA, a secondary BA generated by microbiota, blocks Ca^2+^/NFAT-mediated CD8^+^ T-cell signaling, suppressing effector functions and leading to enhanced tumor progression in colorectal cancer (Cong et al., 2024). Also, the upregulation of BA synthesis in a liver cancer model driven by SV40 large TAG resulted in conjugation of BAs, including TCDCA and LCA. In that case, high TCDCA levels caused CD8^+^ T-cell ROS production and death, reducing the efficacy of α-PD-1 therapy. On the other hand, LCA conjugates triggered endoplasmic reticulum stress responses in CD8^+^ T cells, reducing their capacity to inhibit tumor development (Varanasi et al., 2025). On the contrary, in some cases, BAs might exert pro-immunogenic properties. For instance, secondary BAs were found to impair antitumor immunity via inhibiting effector functions of CD8^+^ T cells in B16 liver tumors; however, primary BAs can increase the expression of CXCL16 on hepatic endothelial cells and induce recruitment of CXCR6^+^ NKT cells, promoting antitumor immunity (Ma et al., 2018). Additionally, dietary administration of UDCA led to decreased liver tumor burden (Varanasi et al., 2025). Moreover, microbiota-generated isomers of BAs were shown to inhibit androgen receptor signaling in CD8^+^ T cells, generating a stem cell-like phenotype and enhancing immunotherapy response (Jin et al., 2025). These findings extend this emerging topic and report that TDCA promotes antitumor immune response and is capable of overcoming microbiota disturbance-associated impairment in ICT response. Altogether, these findings suggest that BA effects are likely highly dependent on structure, concentration, and microenvironment. The effect of antibiotics on bacterial BA metabolism modulates cancer progression and therapy responses through the control of immune signaling pathways and metabolic processes. As each type of BA is associated with unique biological effects, maintaining balance in BA composition in bacteria could enhance anti-cancer immunity without causing any side effects of antibiotic use. Of note, the relationship between microbial BA metabolism and tumor progression remains incompletely understood, as individual BAs may exert either tumor-promoting or tumor-suppressive effects depending on the tissue, receptor signaling, and disease context.

### 5.4 Hepatic diseases

The liver is especially sensitive to disturbances in BA metabolism caused by antibiotic treatment owing to its close relationship with the intestine via enterohepatic circulation. Alteration in the composition of bacteria in the gut causes disturbance in BA biotransformation, FXR and TGR5 signaling, BA transport, and intestinal barrier permeability, leading to bile duct damage, fatty liver disease, and hepatitis. In a study, Xiao et al. (2018) found a mechanism by which exposure to antibiotics can result in cholestasis developing in infants suffering from intestinal failure through disruption of gut microbiome balance. They presented evidence that exposure to oral antibiotics such as gentamicin or vancomycin for 2 weeks disrupts BA metabolism, as well as intestinal microbiota balance. While vancomycin affects mostly Gram-positive bacteria, gentamicin acts predominantly on Gram-negative populations. Nevertheless, gentamicin shows a stronger effect on preservation of BA homeostasis. In both cases, FXR signaling turns out to be one of the pathways contributing to dysregulated BA metabolism under the influence of antibiotics (Xiao et al., 2018). Both antibiotics cause a marked decrease in bacterial colonization of mouse colonic contents. Strikingly, both antibiotics are capable of affecting numerous microorganisms responsible for BA transformation, including Gram-positive *Clostridium* and *Bifidobacterium* and Gram-negative *Eubacterium*. Corresponding to depletion of these microorganisms responsible for BA metabolism, both gentamicin and vancomycin cause high levels of primary BAs in blood and low levels of secondary BAs (Xiao et al., 2018). Furthermore, Xiao et al. (2018) showed that both gentamicin and vancomycin lead to higher content of tauro-β-muricholic acid (TβMCA) in the colonic contents of experimental animals. As a result of these changes, the activity of ileal FXR is inhibited, resulting in lower expression of fibroblast growth factor 15 (FGF15). In the liver, inhibition of FXR causes dysregulated BA metabolism via altered expression of such enzymes as Cyp7a1 and Cyp27a1 responsible for BA synthesis. Apart from changes in BA metabolism, both antibiotics affect BA transport processes. First, it has been noticed upregulation of FXR-regulated BA exporters (Mrp3, Mrp4, Mdr1, and Mdr3) in livers of gentamicin-treated mice. However, in the ileum, expression of FXR-target genes Ostα/Ostβ is inhibited by gentamicin and vancomycin, thus reducing the ability of the intestine to absorb BAs (Xiao et al., 2018). Therefore, these findings indicate that antibiotics are potent disruptors of BA homeostasis and intestinal microbiota balance, which affect BA metabolism via FXR signaling.

Kasai et al. (2023) investigated how gut microbiota perturbation, hepatic inflammation, and fibrosis could interact using a novel nonalcoholic steatohepatitis (NASH) mouse model, where metronidazole or vancomycin was administered. Notably, administration of vancomycin, which targets Gram-positive bacteria, aggravated liver damage, steatohepatitis, and progressive fibrotic changes in iHFC-fed TSNO mice, together with an increased number of hepatic F4/80^+^ macrophages. In particular, CD11c^+^ macrophage subpopulations were significantly enriched, forming hCLSs in this model, an effect exacerbated by the administration of vancomycin. Additionally, the CD11c^+^ macrophages were more closely associated with collagen accumulation in the iHFC + vancomycin groups (Kasai et al., 2023). Unlike vancomycin, the impact of metronidazole, known to target anaerobic bacteria, on these parameters was negligible. Furthermore, vancomycin treatment resulted in significant BA profile alteration due to the inhibition of microbial BSH activity, among other bacterial enzymes involved in BA modification, mainly produced by Gram-positive bacteria (e.g., *Lactobacillus*) (Kasai et al., 2023). Given the established role of BA composition in gut barrier integrity preservation, the observed changes are of special importance, since secondary BA has been shown to regulate intestinal permeability, causing “leaky gut” phenotypes (Stenman et al., 2013; Hegyi et al., 2018; Rohr et al., 2020).

According to previous studies, a HFD is capable of inducing selective elevation of primary 12α-hydroxylated BA within the enterohepatic circulation, whereas primary BA usually dominates in the small intestine as opposed to secondary BA dominating in the large intestine (Yoshitsugu et al., 2019; Hori et al., 2020). As was previously observed, in rats supplemented with CA, the administration of vancomycin results in elevated TCA concentration in the ileum, whereas DCA becomes almost undetectable at this location, and CA levels in feces are elevated during vancomycin treatment (Liu et al., 2022a). Altogether, these data correspond to our findings and indicate that vancomycin is capable of inhibiting microbial 7α-dehydroxylation of CA in the large intestine. Notably, it was shown that TCA, but not DCA, leads to selective elevation of intestinal permeability in the ileum (conjugated BA reabsorption takes place at this segment), the effect being greater than that of DCA in the colon (Liu et al., 2022a). Considering these findings, it is suggested that in the presence of iHFC feeding and vancomycin treatment, there is a disruption of gut barrier integrity due to elevated ileal TCA levels, leading to liver inflammation within the enterohepatic circulation pathway in this NASH model. It should be noted that in this model, vancomycin administration caused a pronounced elevation of hepatic TGR5 expression under the conditions of iHFC feeding. In conjunction with the increased infiltration of macrophages, this TGR5 overexpression might be caused by their recruitment to the inflamed liver under diet- and antibiotic-induced metabolic stress conditions. In summary, there is sufficient evidence from current studies to show that the use of antibiotics causes disruption of hepatic BA balance due to alterations of BA metabolism and signals within the gut. This promotes cholestasis, liver inflammation, fibrosis, and metabolic diseases of the liver via the FXR and TGR5 pathways. Although preclinical studies strongly support a role for altered BA in liver injury, clinical evidence remains limited, highlighting the need for large prospective studies to validate these mechanistic findings in humans.

## 6 Bile acid restoration following antibiotic therapy

With regard to the significance of bacterial BA metabolism in preserving homeostasis in the host organism, much interest has been paid to therapies that can help restore the BA profile following dysbiosis caused by antibiotics (Niculescu et al., 2026). Such approaches as FMT, probiotic administration, antibiotics for preservation of microbiota, and directed microbial treatments can be used to restore BA-transforming bacteria and the pathways of BA signaling (Seekatz et al., 2018; Liu et al., 2025; Yi et al., 2025). FMT is able to restore a normal pattern of fecal BAs more quickly than any other treatment option (Rode et al., 2026). Moreover, the composition of BAs in stool samples of potential donors can serve as a biomarker of FMT donor suitability. As expected, patients suffering from recurrent CDI showed a significant difference in the fecal BA pattern compared with healthy donors at baseline in terms of high primary BAs. CA, CDCA, glycocholic acid (GCA), and glycochenodeoxycholic acid (GCDCA) levels were high. Interestingly, the levels of CDCA were higher but still far from the levels of CA (Rode et al., 2026). Unexpectedly, TCA known for promoting germination of *C. difficile* spores, was present at low levels in this population. The total concentration of fecal BAs was approximately three times higher in patients with recurrent CDI compared with healthy individuals at baseline. Excessively high levels of primary BAs may themselves contribute to the development of diarrhea by affecting colonic motility and secretion, even in the absence of *C. difficile* (Rode et al., 2026). These effects are characteristic of patients suffering from BA diarrhea, some patients with diarrhea-predominant irritable bowel syndrome (IBS-D), and healthy individuals receiving primary BAs (Mottacki et al., 2016). Another study noted an increase in BAs after FMT (Brown et al., 2018). At the same time, other researchers discovered increased levels of FGF19 in the post-treatment period, indicating that treatment leads to enhanced negative feedback in BA synthesis (Monaghan et al., 2019). Therefore, low pre-FMT levels of FGF19 in the Rode et al. (2026) cohort may reflect increased BA synthesis in response to disruption of this regulation. Disruption might have occurred due to inflammation or/and *C. difficile* itself or both (Monaghan et al., 2019). Besides, increased intestinal transit time associated with diarrhea could impair reabsorption of BAs and their biotransformation by gut microbes. In turn, this could contribute to the disruption of the FGF19-BA axis in IBS-D (Duboc et al., 2012; Shin et al., 2013). Fast transition to deconjugated BAs after FMT and rectal bacteriotherapy indicates the ability of the bacterial mixture to provide BSH activity. Further increase in secondary BA levels observed in the FMT group depends on the transfer of bacteria or enzymes needed for 7α-dehydroxylation (Alnouti, 2009). This reaction can occur only if BAs are first deconjugated. In summary, these results indicate that BSH activity alone is sufficient to inhibit germination of *C. difficile* spores and prevent recurrence of infection under laboratory conditions. The transferred mixture seems to be able to restore BSH activity, but slower transformation of BAs into secondary forms can be responsible for less desirable therapeutic effects of rectal bacteriotherapy.

In order to assess the impact of FMT on microbiota-dependent metabolic processes in patients with R-CDI, Weingarden et al. (2014) conducted untargeted metabolomic profiling and quantified concentrations of BAs in pre-FMT and post-FMT stool samples. Their findings indicate that restoration of a healthy microbial community following FMT goes hand-in-hand with a swift normalization of the fecal BA profile. First of all, pronounced dysbiosis identified in pre-FMT samples can be explained by the fact that these patients were receiving antibiotics at the time of sample collection, mainly vancomycin. Considering the diversity of roles that colonizing microbiota play in metabolism, a comprehensive characterization of the metabolic effects of FMT was attempted using both targeted and untargeted metabolomics (Weingarden et al., 2014). Indeed, post-FMT samples demonstrated similar metabolic characteristics to healthy donors’ stools and a marked divergence from pre-FMT samples. Within the whole group of metabolites analyzed, BAs and bile salts appeared to be the largest contributors to separation between pre- and post-FMT samples, followed by donors’ stools (Weingarden et al., 2014). Hence, it can be concluded that BA remodeling is one of the main metabolic changes caused by FMT. Another interesting observation was related to the lack of secondary BAs in all pre-FMT samples. Taking into account the crucial role of the gut microbiota in BA metabolism, the absence of secondary BAs indicates a severe loss of BA-metabolizing microorganisms, most likely due to continuous exposure to antibiotics (Hashimoto et al., 1996; Carman et al., 2005). After FMT, it appears that such metabolic activities return, which might be explained by the re-establishment of relevant microbial populations in patients with R-CDI. Restoration of BA metabolism can potentially be used to explain several clinical observations, including persistent mild diarrhea in patients receiving suppressive doses of vancomycin prior to FMT (Weingarden et al., 2014). Indeed, high primary BA content in feces is often associated with diarrhea, while the opposite case occurs when the concentration of secondary BAs exceeds that of primary ones (Shin et al., 2013). Predominantly high content of primary BAs and their conjugates in the stools of R-CDI patients suggests their disease relapse during antibiotic withdrawal. In turn, fast restoration of the balance between BA groups after FMT corresponds to the typical CDI relapse period – 2–3 weeks after discontinuation of antibiotic therapy. Based on these findings, it can be concluded that changes in BA composition and concentration are mediated by the alteration of fecal microbiota after FMT. Most likely, FMT restores the population of microorganisms responsible for secondary BA synthesis and leads to the inhibition of *C. difficile* spore germination and toxin production. Thus, restoration of the intestinal microflora and subsequent restoration of BA balance led to inhibition of *C. difficile* growth and establishment of favorable conditions for patients’ recovery.

The possible restoration of microbial BAs’ biosynthesis via the FXR/TGR5 pathway will assist in creating a balance between signals, which can lead to the maintenance of proper mucosal immune homeostasis (Baars et al., 2015; Pathak et al., 2018). At the same time, disruptions found in linoleic acid and α-linolenic acid metabolism in the case of antibiotic-associated diarrhea (AAD) have been related to the decrease in the activity of peroxisome proliferator-activated receptors (PPARs) as well as increased inflammation in the intestine (Yuan et al., 2015; Ungaro et al., 2017). Supplementation with RH2 could be used to overcome metabolic imbalances and stimulate the coordination in the recovery of microbial and host metabolic pathways. The results found seem to fit the previously mentioned protective mechanisms against antibiotic-induced epithelial damage (Duan et al., 2022). In summary, the application of metabolomic approaches demonstrates that the use of RH2 can contribute to the recovery of host metabolism and intestinal barrier function. Thus, all the above-mentioned results prove the possibility of using *Clostridium butyricum* RH2 as an effective treatment for children who experience antibiotic-associated diarrhea (AAD). It is possible to conclude that *C. butyricum* RH2 helps partially restore microbiota, metabolic homeostasis, and epithelial structure even during antibiotic therapy. Nevertheless, further investigation into these mechanisms should be conducted.

Exposure to antimicrobial agents results in a significant reduction in the ability of the microbiota to carry out the BA transformation process, an effect which progressively recovers toward normalcy after antibiotic treatment is discontinued (Kelly et al., 2019). The perturbation in the transformation process results in an elevation in the concentration and fraction of CA in feces. *Saccharomyces boulardii* administered together with and after antibiotics partially rescued the decrease in the BA transformation process and reduced the subsequent increase in fecal CA content over a long period (Kelly et al., 2019). In this case, Kelly et al. (2019) induced a controlled dysbiosis state using the antibiotic treatment. A similar pattern of perturbation in BA metabolism has been observed in IBD conditions. Reduction in microbiota activity concerning BA transformations and changes in BA deconjugation have also been observed in this condition (Duboc et al., 2013). In Kelly et al. (2019) study, however, there were no changes either in the fraction or concentration of the amino-conjugated BAs at all time points. The concentration of amino-conjugated BAs was less than 4% of the total fecal BA pool at all time points. The observation that fecal BA composition changes after exposure to antibiotics indicates that BA metabolism can be affected by changes in the microbiota population (Duboc et al., 2013). The expanding understanding of BA signaling has accelerated the development of translational strategies aimed at restoring microbiota-derived BA homeostasis after antibiotic exposure. Pharmacological modulation of BA receptors, particularly FXR and TGR5, has emerged as a promising approach to regulate BA synthesis, glucose and lipid metabolism, inflammatory responses, and intestinal barrier integrity. In parallel, microbiota restoration strategies–including probiotics, prebiotics, synbiotics, postbiotics, and FMT–seek to replenish BSH-producing and 7α-dehydroxylating bacteria, thereby promoting recovery of the secondary BA pool and re-establishing host–microbiota signaling. Several microbiota-based therapies have already demonstrated clinical efficacy, most notably FMT for recurrent CDI, while BA receptor agonists and engineered microbiome-based therapeutics are being actively evaluated for metabolic, inflammatory, and liver diseases. Collectively, these advances underscore the translational potential of targeting the antibiotic–microbiota–BA axis as a precision therapeutic strategy to preserve host homeostasis and improve clinical outcomes.

## 7 Current controversies, limitations, and challenges

Despite substantial advances in understanding the antibiotic–microbiota–BA axis, several important challenges remain. Although numerous studies consistently demonstrate that antibiotics alter microbial BA metabolism, the magnitude and direction of these effects vary considerably among studies. These discrepancies likely reflect differences in antibiotic class, treatment duration, host genetics, dietary composition, baseline microbiota structure, analytical methodologies, and disease models. Consequently, direct comparisons across studies remain difficult, and caution should be exercised when extrapolating findings from one experimental setting to another. Conflicting observations are particularly evident regarding the biological functions of individual secondary bile acids such as DCA. While physiological concentrations of DCA contribute to intestinal homeostasis and colonization resistance against enteric pathogens, sustained elevations under pathological conditions have been associated with chronic inflammation, DNA damage, and tumor promotion (Keating et al., 2009; Tsuei et al., 2014). These apparently contradictory findings emphasize that BA signaling is highly context-dependent and influenced by tissue specificity, receptor expression, metabolic state, and disease stage (Wolf et al., 2026). Another important limitation of the current literature is the predominance of preclinical animal studies, whereas longitudinal human studies integrating microbiome composition, BA metabolomics, host transcriptomics, and clinical outcomes remain relatively scarce (Zacharias et al., 2022; Barragan-Galvez et al., 2026). Furthermore, considerable heterogeneity exists in bile acid analytical platforms, microbiome sequencing approaches, and bioinformatic pipelines, limiting reproducibility and cross-study comparisons (Hu et al., 2023). Future investigations should prioritize standardized methodologies, integrated multi-omics analyses, and well-designed prospective clinical studies to establish causal relationships and facilitate the translation of microbiome-targeted interventions into clinical practice.

Despite the accumulating evidence demonstrating that exposure to low-level concentrations of antibiotics could disturb the balance in BA metabolism due to their impact on the composition of the gut microbiome, there are still some difficulties that need to be addressed. The important one is associated with the uncertainty regarding the adequacy of the currently set safety threshold (Wang et al., 2024). Although the changes in BA metabolism have already been identified at the exposure levels that are orders of magnitude lower than those defined as safe, it is still unknown whether these alterations might have a significant clinical effect on the organism. Another difficulty lies in the nature of human studies being predominantly observational and cross-sectional and therefore failing to draw a causal conclusion and raising the issue of reverse causality (Wang et al., 2024). Besides, a single measurement of urinary BA profile may not serve as a good marker of long-term exposure to antibiotics because of the short biological half-life of the majority of antibiotics and sporadic exposure to these compounds in food, water, and medications. The absence of gut microbiome analysis while conducting the study is also one of the limitations. Besides, there is a difficulty associated with a non-monotonic (U-shaped) dose-response relationship between antibiotic concentration and the changes in BA metabolism, making conventional risk assessment almost impossible (Wang et al., 2024). In addition, the considerable variability in response to antibiotics that can result from genetic predisposition, different diets, etc. complicates the identification of common biomarkers of BA disturbances.

## 8 Concluding remarks and future directions

In this review, we present a systematic overview of the mounting evidence that antibiotics regulate BA metabolism through the profound impact of the administered antibiotics on the gut microbiota. Contrary to earlier reviews that mainly covered the overall role of the gut–bile axis, we particularly stress the molecular mechanisms of antibiotic-induced dysbiosis-mediated changes in microbial BA transformation, host signaling, and the pathogenesis of diseases. Overall, current research clearly shows that the loss of BA-transforming microbes caused by antibiotic use results in a decrease in BSH activity, secondary BA synthesis, and disturbance of enterohepatic signaling via FXR, TGR5, NLRP3 inflammasome, GLP-1, and mTOR pathways. The consequences of such dysregulation include not only changes in intestinal functions but also metabolic regulation, immune homeostasis, liver performance, neurodegenerative diseases, oncogenesis, and susceptibility to infections. Furthermore, the recently developed microbiota-targeted therapies, which consist of microbiota-preserving antibiotics, probiotics, FMT, and manipulation of microbial BA metabolism, demonstrate the therapeutic potential of preservation or recovery of BA homeostasis under antibiotic treatment.

Despite the promising therapeutic potential of restoring BA metabolism, several important safety considerations should be acknowledged. BAs, particularly DCA, exhibit concentration- and context-dependent biological activities, and chronic or excessive exposure has been associated with epithelial cytotoxicity, oxidative DNA damage, barrier dysfunction, chronic inflammation, and increased risk of gastrointestinal and hepatobiliary carcinogenesis. Likewise, although TDCA and other secondary BAs have demonstrated immunomodulatory properties in preclinical studies, their long-term safety, optimal dosing, tissue-specific distribution, and off-target effects remain insufficiently characterized. Similarly, while FMT effectively restores microbial BA metabolism, concerns remain regarding donor variability, transmission of opportunistic pathogens, unintended metabolic alterations, and variable long-term efficacy. Therefore, future therapeutic strategies should prioritize precision modulation of microbial BA metabolism, selective targeting of BA receptors, engineered microbial consortia, and personalized microbiome-based interventions rather than indiscriminate supplementation with BAs. Such approaches may maximize therapeutic benefit while minimizing potential toxicological risks.

Future research should move beyond descriptive characterization of antibiotic-induced microbiota alterations toward mechanistic and translational investigations that integrate metagenomics, metabolomics, transcriptomics, proteomics, and spatially resolved single-cell technologies. Such approaches will improve our understanding of how antibiotic-induced perturbations of microbial BA metabolism influence tissue-specific signaling networks and disease susceptibility. Equally important is the development of standardized analytical platforms and large prospective clinical studies to identify robust biomarkers of BA dysregulation and validate causal mechanisms in human populations. From a clinical perspective, preserving or restoring microbiota-derived BA homeostasis represents a promising strategy for minimizing the unintended consequences of antibiotic therapy. Emerging interventions–including microbiota-preserving antibiotics, live biotherapeutic products, engineered BA-producing bacteria, fecal microbiota transplantation, and selective modulation of FXR- and TGR5-dependent signaling–have the potential to transform the management of metabolic, inflammatory, infectious, hepatic, neurological, and malignant diseases. Ultimately, integrating microbiome profiling with BA metabolomics and host molecular signatures may enable precision medicine approaches that optimize antibiotic use while maintaining the beneficial functions of the gut microbiota and its BA metabolites.
